# English version of Japanese Clinical Practice Guidelines 2022 for gastrointestinal stromal tumor (GIST) issued by the Japan Society of Clinical Oncology

**DOI:** 10.1007/s10147-024-02488-1

**Published:** 2024-04-13

**Authors:** Seiichi Hirota, Ukihide Tateishi, Yuji Nakamoto, Hidetaka Yamamoto, Shinji Sakurai, Hirotoshi Kikuchi, Tatsuo Kanda, Yukinori Kurokawa, Haruhiko Cho, Toshirou Nishida, Akira Sawaki, Masato Ozaka, Yoshito Komatsu, Yoichi Naito, Yoshitaka Honma, Fumiaki Takahashi, Hironobu Hashimoto, Midori Udo, Minako Araki, Sumito Nishidate, Members of the Systematic Review Team of the Present Guidelines

**Affiliations:** 1 Department of Surgical Pathology, Hyogo Medical University School of Medicine, Nishinomiya, Japan; 2 Department of Diagnostic Radiology and Nuclear Medicine, Tokyo Medical and Dental University, Tokyo, Japan; 3 Department of Diagnostic Imaging and Nuclear Medicine, Graduate School of Medicine, Kyoto University, Kyoto, Japan; 4 Department of Pathology, Graduate School of Medicine, Dentistry and Pharmaceutical Sciences, Okayama University, Okayama, Japan; 5 Department of Diagnostic Pathology, Japan Community Healthcare Organization Gunma Central Hospital, Maebashi, Japan; 6 Department of Surgery, Hamamatsu University School of Medicine, Hamamatsu, Japan; 7 Department of Gastroenterology, Southern TOHOKU General Hospital, Koriyama, Japan; 8 Department of Gastroenterological Surgery, Graduate School of Medicine, Osaka University, Suita, Japan; 9 Department of Surgery, Tokyo Metropolitan Cancer and Infectious Diseases Center Komagome Hospital, Tokyo, Japan; 10 Department of Surgery, Japan Community Healthcare Organization Osaka Hospital, Osaka, Japan; 11 Department of Medical Oncology, Shonan Kamakura General Hospital, Kamakura, Japan; 12 Department of Hepato-Biliary-Pancreatic Medicine, Cancer Institute Hospital of Japanese Foundation for Cancer Research, Tokyo, Japan; 13 Department of Cancer Chemotherapy, Hokkaido University Hospital Cancer Center, Sapporo, Japan; 14 Department of General Internal Medicine, National Cancer Center Hospital East, Kashiwa, Japan; 15 Department of Head and Neck, Esophageal Medical Oncology, National Cancer Center Hospital, Tokyo, Japan; 16 Department of Information Science, Iwate Medical University, Morioka, Japan; 17 Department of Pharmacy, National Cancer Center Hospital, Tokyo, Japan; 18 Nursing Department, Osaka Police Hospital, Osaka, Japan; 19 Association of Chubu GIST Patients and Their Families, Nagoya, Japan; 20 Specified Nonprofit Corporation GISTERS, Kamakura, Japan

**Keywords:** Gastrointestinal stromal tumor (GIST), Clinical practice guidelines, Minds manual for guideline development, Expert consensus

## Abstract

The Japan Society of Clinical Oncology Clinical Practice Guidelines 2022 for gastrointestinal stromal tumor (GIST) have been published in accordance with the Minds Manual for Guideline Development 2014 and 2017. A specialized team independent of the working group for the revision performed a systematic review. Since GIST is a rare type of tumor, clinical evidence is not sufficient to answer several clinical and background questions. Thus, in these guidelines, we considered that consensus among the experts who manage GIST, the balance between benefits and harms, patients’ wishes, medical economic perspective, etc. are important considerations in addition to the evidence. Although guidelines for the treatment of GIST have also been published by the National Comprehensive Cancer Network (NCCN) and the European Society for Medical Oncology (ESMO), there are some differences between the treatments proposed in those guidelines and the treatments in the present guidelines because of the differences in health insurance systems among countries.

**Supplementary Information:**

The online version contains supplementary material available at 10.1007/s10147-024-02488-1.

## Introduction

### Purpose of these guidelines

The aim of these guidelines is to improve the prognoses of the patients with gastrointestinal stromal tumor (GIST) through good medical practice by providing appropriate treatment policies for non-expert clinicians who do not have enough experience in the treatment of GIST, a rare tumor type. Thus, the main users of these guidelines are non-expert clinicians who are involved in managing GIST. These guidelines also provide information on the management of GIST to medical personnel other than doctors, and to GIST patients and their families. While the target patients of these guidelines are those with GIST having various mechanisms of tumorigenesis and belonging to all age groups, the chief target age group is adults, because most cases of GIST occur in adults. Therefore, the descriptions in these guidelines need to be applied carefully to juvenile patients. These guidelines provide the policies for the standard management of GIST covered by Japanese insurance, but do not restrict the physician’s treatment policies or discretion. Therapies other than those described in these guidelines are possible according to the patients’ wishes and status of the facilities. The contents of these guidelines have not been prepared as reference materials for medical lawsuits.

### Revision methods

The present guidelines are a revised version of the previous guidelines and include new evidence. They were revised in accordance with the Minds Manual for Guideline Development 2014 and 2017. The scope of the guidelines including the basic policy for this revised version was defined, and approved by the working group (WG). It included a plan to revise old algorithms to create new ones and shows the corresponding portions of clinical questions (CQs) and background questions (BQs). The CQs in the previous version were re-evaluated, and revised to create new ones. Some previous CQs were positioned as BQs when the contents were considered to have become common knowledge. Closed questions were adopted in most of the present CQs and BQs. A literature search using PubMed and the Cochrane Library was performed by the Japan Medical Library Association. Since the accumulation of evidence was insufficient due to the scarcity of high-quality articles in this field, observation study papers were incorporated wherever possible. A systematic review (SR) team independent of the WG conducted an SR with screening conducted twice. After the evaluation of the “body of evidence” was completed depending on the evidence evaluation of individual studies, the SR report was completed. According to the report, each designated member of the WG prepared a draft considering the strength of the evidence (certainty) (Table 1), balance of benefits and harms, patients’ wishes and social medical expenses. The drafts were discussed and voted on by all members of the WG using the GRADE Grid system. When the approval rate by vote was 80% or more, the strength of the evidence was determined. When the approval rate by vote was less than 80%, a second vote was taken following discussion. In the case where the approval rate was less than 80% even after the second vote, the strength of the evidence was classified as “Not Graded.” Recommendations were expressed by combining “directions of recommendation” and “strength of recommendation” (Tables 2 and 3). For some BQs, only the content approval was done because they were not closed questions on medical practice.

**Table 1:** Quality of evidence and definitions

A (High quality)	Further research is very unlikely to change our confidence in the estimate of effect
B (Moderate quality)	Further research is likely to have an important impact on our confidence in the estimate of effect and may change the estimate
C (Low quality)	Further research is very likely to have an important impact on our confidence in the estimate of effect and is likely to change the estimate
D (Very low quality)	Any estimate of effect is very uncertain

**Table 2:** Strength of recommendation

	Strength of recommendation	
	Strong	Weak
Direction of recommendations		
For	We recommend…	We suggest…
Against	We recommend not…	We suggest not…

**Table 3:** Strength of recommendation and quality of evidence

Strength of recommendation	Quality of evidence
1 (Strong recommendation)	A (High quality)
2 (Weak recommendation)	B (Moderate quality)
	C (Low quality)
	D (Very low quality)

### External evaluation

External evaluation was performed by the evaluation working group for the revision of the GIST practice guidelines, public comments were made by members of the Japan Society of Clinical Oncology, and the AGREE II evaluation was conducted by the evaluation committee of the Clinical Practice Guidelines in the Japan Society of Clinical Oncology. The WG discussed the comments from the external evaluation and responded to them.

### Algorithms and supplement

Algorithms and supplemental algorithms for GIST diagnosis and treatment adopted in the present guideline are as follows.

**Fig. 1:**
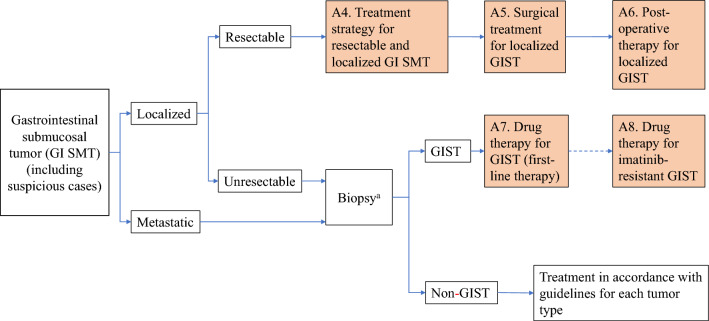
Algorithm 1, Outline of diagnosis and therapy for gastrointestinal submucosal tumors. **a** Methods to obtain the tissue are not restricted. They include percutaneous needle biopsy and biopsy at exploratory laparotomy

**Fig. 2:**
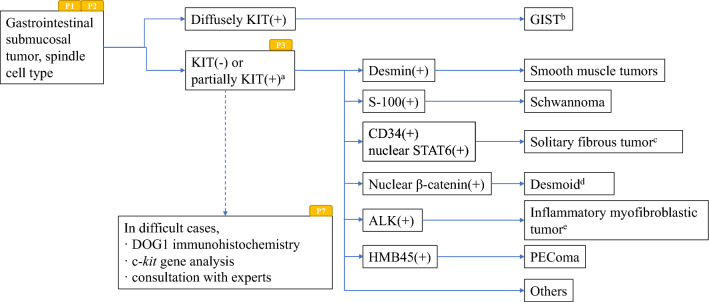
Algorithm 2, Differential diagnosis of spindle cell type GIST. **a** Most of the spindle cell type GISTs are diffusely positive for KIT, and KIT-negative and partially KIT-positive spindle cell type GISTs are very rare. Tumors with partial KIT-positivity should be considered non GISTs with nonspecific KIT staining. **b** Histological findings of tumors with HE staining have to be consistent with those of GIST. **c** Confirmation of the presence of NAB2-STAT6 fusion gene is recommended. **d** Mutational analysis of CTNNB1 gene encoding beta-catenin is recommended. **e** Analysis of ALK fusion gene by PCR or FISH is recommended. P1, P2, P3 and P7 mean “see Pathology BQ1, BQ2, BQ3 and BQ7”, respectively

**Fig. 3:**
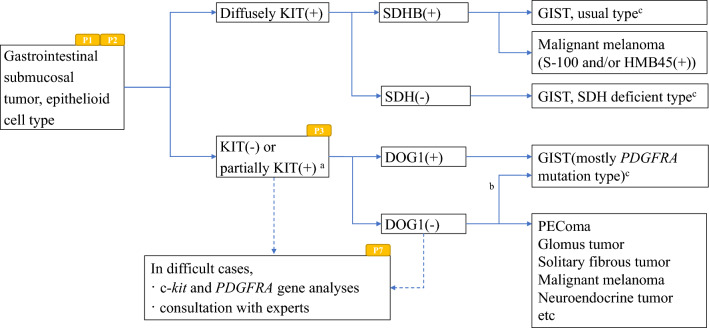
Algorithm 3, Differential diagnosis of epithelioid cell type GIST. **a** Partially KIT-positive tumors should be considered non GISTs showing nonspecific KIT staining. **b** Because of rarity of DOG1-negative GISTs, gene analysis should be performed for those tumors especially PDGFRA gene. **c** Histological findings of the tumor with HE staining have to be consistent with those of GIST. P1, P2, P3 and P7 mean “see Pathology BQ1, BQ2, BQ3 and BQ7”, respectively

**Fig. 4:**
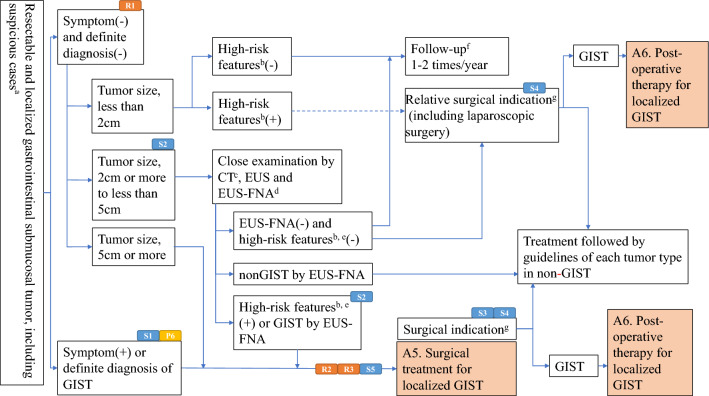
Algorithm 4, Treatment strategy for resectable and localized gastrointestinal submucosal tumors. **a** Epithelial tumors have to be excluded by biopsy under endoscopy. Biopsy from the serosal side is prohibited. **b** Findings of ulcer formation, irregular margin, and enlargement are included. **c** Enhanced CT (oral or transvenous) with continuous slice 5-mm thick or less is recommended. **d** EUS-FNA is recommended but not necessary. **e** Findings of necrosis, hemorrhage, irregular margin, and heterogeneity by enhanced CT and those of heterogeneity, irregular margin, and lymph node enlargement by EUS are included. **f** Follow-up by endoscopy including EUS is recommended. **g** Intraoperative pathological examination is recommended when a preoperative pathological diagnosis is not made. R1, R2, R3, P6, S1, S2, S3, S4 and S5 mean “see Radiology BQ1, BQ2, BQ3, Pathology BQ6, Surgery CQ1, CQ2, CQ3, BQ4 and CQ5”, respectively

**Fig. 5:**
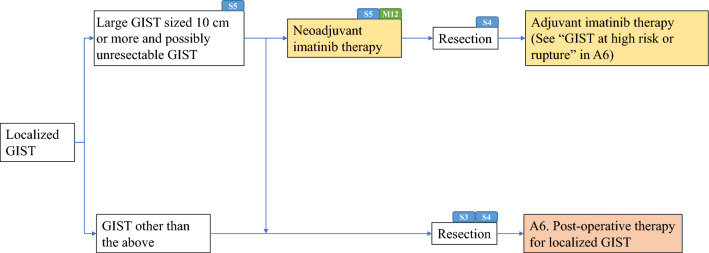
Algorithm 5, Surgical treatment for localized GIST. S3, S4, S5 and M12 mean “see Surgery CQ3, BQ4, CQ5 and Medicine CQ12”, respectively

**Fig. 6:**
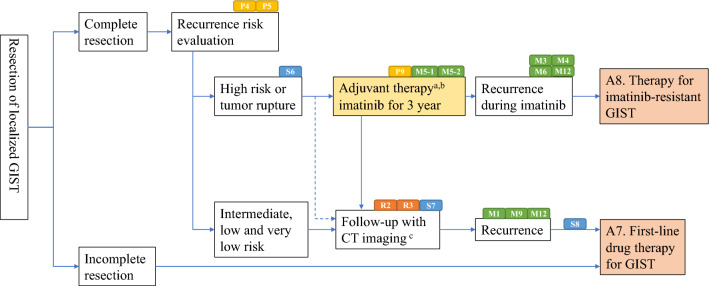
Algorithm 6, Post-operative therapy for localized GIST. **a** Efficacy of adjuvant imatinib therapy is not established in GISTs with low and intermediate risk of recurrence. **b** Follow-up by enhanced CT is usually carried out every 6 months (evidence unknown). **c** Follow-up by enhanced CT is usually carried out every 4–6 months in the case of GISTs with high risk of recurrence and/or tumor rupture and every 6–12 months in the case of GISTs with very low, low, and intermediate risk of recurrence (evidence unknown). R2, R3, P4, P5, P9, S6, S7, S8, M1, M3, M4, M5-1, M5-2. M6, M9 and M12 mean “see Radiology BQ2, BQ3, Pathology BQ4, BQ5, BQ9, Surgery CQ6, BQ7, CQ8, Medicine CQ1, CQ3, CQ4, BQ5-1, CQ5-2, BQ6, CQ9 and CQ12”, respectively

**Fig. 7:**
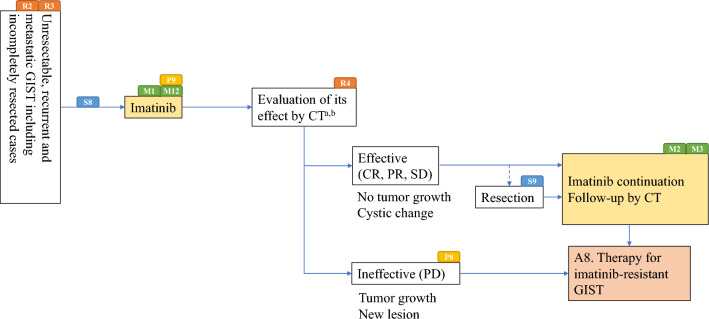
Algorithm 7, First-line drug therapy for GIST. **a** Follow-up by enhanced CT is usually carried out every 4–6 months (evidence unknown). **b** Efficacy of FDG-PET/CT has been reported, but it is not covered by insurance. R2, R3, R4, P8, P9, S8, S9, M1, M2, M3 and M12 mean “see Radiology BQ2, BQ3, CQ4, Pathology BQ8, BQ9, Surgery CQ8, CQ9, Medicine CQ1, BQ2, CQ3, and CQ12”, respectively

**Fig. 8:**
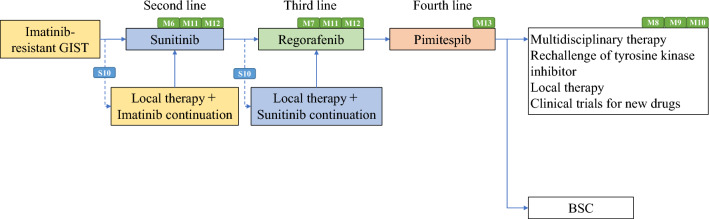
Algorithm 8, Therapy for imatinib-resistant GIST. S10, M6, M7, M8, M9, M10, M11 and M12 mean “see Surgery CQ10, Medicine BQ6, BQ7, CQ8, CQ9, CQ10, CQ11 and CQ12”, respectively

- Algorithm 1 (Fig. 1), Outline of diagnosis and therapy for gastrointestinal submucosal tumors
- Algorithm 2 (Fig. 2), Differential diagnosis of spindle cell type GIST
- Algorithm 3 (Fig. 3), Differential diagnosis of epithelioid cell type GIST
- Algorithm 4 (Fig. 4), Treatment strategy for resectable and localized gastrointestinal submucosal tumors
- Algorithm 5 (Fig. 5), Surgical treatment for localized GIST
- Algorithm 6 (Fig. 6), Post-operative therapy for localized GIST
- Algorithm 7 (Fig. 7), First-line drug therapy for GIST
- Algorithm 8 (Fig. 8), Therapy for imatinib-resistant GIST
- Supplemental Algorithm 1 (Fig. S1), Genotype of GIST
- Supplemental Algorithm 2 (Fig. S2), Differential diagnosis for multiple GISTs

## Radiological diagnosis part

### Overview of the radiological diagnosis part

#### Diagnostic imaging useful for diagnosing submucosal tumors

##### Lesions less than 2 cm in diameter

When submucosal tumors (SMTs) are suspected on screening radiography and endoscopy, endoscopic biopsy is mandatory. In addition, the tumor diameter is a criterion for determining the treatment policy. Therefore, measurement of tumor diameter is performed. SMTs less than 2 cm in diameter featuring hemispherical, smooth outline, non-ulcerated, or non-depressed are to be followed up once or twice annually.

##### Lesions larger than 2 cm in diameter

If the tumor diameter is 2 cm or more but less than 5 cm, has irregular margins, ulceration or pitting, or is enlarged, further examination by computed tomography (CT), endoscopic ultrasonography (EUS), and endoscopic ultrasonography-fine needle aspiration (EUS–FNA) biopsy should be performed. Staging by surgical excision is considered for lesions with a diameter of 5.1 cm or more, symptomatic cases, or GIST diagnosed by biopsy.

Continuous CT slice thickness and intervals of 5 mm or less are standard, but 3D data with slice thickness and intervals of 2 mm or less are desirable. Scan range includes the upper abdomen to the pelvis for staging (to detect intraperitoneal dissemination or ascites) with oral contrast medium and intravenous contrast-enhanced CT is required. Portal phase CT is recommended for single scan. However, for more accurate evaluation of liver metastases, multiphase CT including pre-contrast, arterial, portal, and delayed scan is recommended. Oral contrast medium should be added to water or an effervescent agent as appropriate for the purpose of improving observation. If contrast-enhanced CT cannot be performed due to allergies, etc., or if it is difficult to determine the lesion even with contrast-enhanced CT, magnetic resonance imaging (MRI) is to be performed. It can be expected that diffusion-weighted imaging will detect peritoneal disseminated lesions. When diagnosis by the above imaging tests is difficult, ^18^F-2-fluoro-2-deoxy-D-glucose positron emission tomography (FDG-PET)/CT is to be performed. FDG-PET/CT is useful for diagnosing peritoneal disseminations and unexpected distant metastases.

#### Diagnostic imaging useful for determining the effect of drug therapy

##### Gastrointestinal imaging, endoscopy, and ultrasonography

Gastrointestinal imaging and endoscopy can reveal changes in tumor size and shape, but internal changes are not often determined. Ultrasonography is radiation-free and can be easily repeated, and makes it possible to evaluate drug efficacy based on size change. However, a method to quantify efficacy is yet to be established.

##### Contrast-enhanced CT and MRI

The NCCN clinical practice guidelines and the ESMO consensus report recommend use of contrast-enhanced CT to measure the change in tumor size [1, 2]. CT value is measured and quantified for efficacy determination since there are many cases in which the therapeutic effect is obtained with decreased blood flow and cystic formation within the tumor. Tumor size reduction greater than or equal to 10% or decrease of CT value greater than or equal to 15% is considered partial remission (PR) (Table 4) [3]. The CT findings of GIST often change rapidly 1–2 months after starting drug therapy. In addition, rapid tumor growth may occur with relapse. The optimal CT interval is every 1–2 months after initiation of drug therapy and thereafter every 3–6 months unless there were changes in imaging findings or symptoms. When findings indicative of recurrence are observed, it is considered advisable to shorten the interval to every 1–2 months. Although MRI enables changes in size, internal structure, and blood flow to be monitored, the usefulness of MRI encompassing CT is not clear for evaluating therapeutic effect except for non-radiation exposure.

**Table 4:** Modified CT response evaluation criteria

Response	Definition (The sum of longest diameters of target lesions as defined in RECIST 1.1)
CR	Disappearance of all lesions
	No new lesions
PR	A decrease in size of ≥ 10% or a decrease in tumor density (HU) ≥ 15% on CT
	No new lesions
	No obvious progression of nonmeasurable disease
SD	Does not meet the criteria for CR, PR, or PD
	No symptomatic deterioration attributed to tumor progression
PD	An increase in tumor size of ≥ 10% and does not meet criteria of PR by tumor density (HU) on CT
	New lesions

CR, complete response; PR, partial response; HU, Hounsfield unit; CT, computed tomography; SD, stable disease; PD, progressive disease; RECIST, Response Evaluation Criteria in Solid Tumors. Table 1 is adapted from reference 3

##### FDG-PET/CT

It is known that FDG-PET/CT can sensitively reflect changes in metabolism and blood flow caused by drug therapy. The decrease in glucose metabolism occurs early after initiation of treatment and precedes morphological tumor shrinkage. FDG–PET/CT findings change rapidly 1–2 months after starting drug therapy.

The optimal FDG-PET/CT interval for evaluating drug efficacy is at least 10 days because of the flare phenomenon, which shows a temporary increase in metabolic activity due to activated immune cells after the start of drug therapy [1]. FDG–PET/CT can be an early predictor of tumor shrinkage when monitoring drug therapy. However, in Japan, FDG-PET/CT is not covered by health insurance for evaluating drug efficacy in GIST.

### Questions

#### Radiology 1 (BQ): Is EUS-FNA useful for making a definitive diagnosis of GIST?

- **Recommendation:** We suggest that EUS-FNA is performed to make a definitive diagnosis of GIST.
- **Strength of recommendation:** 2 (Weak recommendation)
- **Quality of evidence:** B (Moderate quality)
- **Consensus rate:** 100%

Pathologic diagnosis using forceps biopsy, EUS-FNA, incisional mucosal biopsy, and boring biopsy is essential for making a definitive diagnosis of GIST. When the mucous membrane is lost and the tumor is exposed, tumor tissue can be collected with normal biopsy forceps. However, if the tumor is not exposed, EUS-FNA biopsy or mucosal incision-assisted biopsy is required. EUS–FNA biopsy can provide specimens sufficient for diagnosis with few complications. Therefore, definitive diagnosis of GIST can almost always be made by EUS-FNA biopsy in combination with immunohistochemical staining. However, there is no evidence regarding the usefulness of EUS-FNA biopsy in the definitive diagnosis of patients with suspected GIST.

As a result of a qualitative systematic review, the diagnostic indicators differed among studies, but the accuracy rate reported in all studies was 62.5–97% for the cohort and 61.6–100% for case–control studies, respectively [4–19].

EUS-FNA is considered useful for making a definitive diagnosis of GIST, but its effectiveness compared with other diagnostic methods is not clear. Efficacy and safety have not been sufficiently investigated, and there may be bias in subjects, operators, facilities, etc.

In addition, EUS-FNA is not an examination that can be easily performed like general endoscopy. Although it is covered by insurance, few facilities in Japan are equipped with the convex ultrasound endoscopes used in EUS–FNA. Based on these findings, EUS-FNA is optional because it provides a definitive diagnosis. However, it should be selected only after making a comprehensive assessment including clinical usefulness.

#### Radiology 2 (BQ): Are CT and MRI useful for determination of the clinical staging and recurrence of GIST?

- **Recommendation:** We recommend that CT and MR images are taken for determination of the clinical staging and recurrence of GIST.
- **Strength of recommendation:** 1 (Strong recommendation)
- **Quality of evidence:** B (Moderate quality)
- **Consensus rate:** 82.4%

CT, especially contrast-enhanced CT of the trunk, is usually used for staging and re-staging of GIST. MRI is also performed in cases in which iodine contrast media is contraindicated or when CT findings are inconclusive. Although there is no direct evidence for this BQ, CT and MRI are routinely used in patients with GISTs that require staging and re-staging in clinical practice, and no alternative method has been established; CT and MRI are positioned as the standard. Therefore, CT and MRI are strongly recommended as modalities to be used when staging or re-staging is necessary for GIST patients [20–26].

It should be noted, however, that the need for staging or re-staging in individual cases is a matter of debate and is not covered in this BQ. It goes without saying that the indication for testing should be determined according to the risk–benefit ratio. As for periodic postoperative follow-up of asymptomatic patients for early diagnosis of recurrence, there are no established criteria or methods for postoperative surveillance backed by a sufficient scientific rationale, and no reports show a contribution to improving survival. Future studies are warranted.

#### Radiology 3 (BQ): Is FDG-PET useful for determination of the clinical staging and recurrence of GIST?

- **Recommendation:** We suggest that FDG-PET is performed for determination of the clinical staging and recurrence of GIST.
- **Strength of recommendation:** 2 (Weak recommendation)
- **Quality of evidence:** C (Low quality)
- **Consensus rate:** 94.1%

No literature verifies the usefulness of FDG-PET/CT in staging and re-staging in terms of prognosis improvement; however, there is a report demonstrating the good diagnostic performance of lesion detection compared with conventional methods such as CT. Gayed et al. reported that CT had a sensitivity of 93% and specificity of 100%, and FDG-PET had a sensitivity of 86% and specificity of 98%, with no statistically significant difference between the two methods for GIST staging in 54 patients with 122 lesions [27]. The tendency of lesions to produce false negative results differed between CT and FDG-PET, with small lesions in the liver, lung, and peritoneum being false negative in FDG-PET and lesions in bone (flat bone) being false negative in CT. In a Japanese multicenter study of 41 cases of GIST, Kaneta et al. reported that peritoneal dissemination was newly detected by FDG-PET in one of eight patients who were imaged for staging purposes, and metastasis (liver, bone, intestine) was newly seen in two of 33 patients who were diagnosed with recurrence [28]. In addition, two patients had false-negative staging (gastric and small intestinal lesions), two patients had false-negative recurrence (small liver metastases), and one patient had false-positive recurrence (esophageal metastasis).

FDG-PET/CT tends to give false-negative results for small metastases due to its limited spatial resolution. Still, there is no significant difference in its diagnostic performance for staging and re-staging compared to CT, and it can be used for staging and recurrence diagnosis. In routine clinical practice, some institutions perform FDG-PET/CT when there is some doubt about the diagnostic decision made based on CT or MRI. In addition, there are reports that FDG accumulation in GISTs is associated with malignancy and prognosis, and FDG-PET/CT may provide additional qualitative information about the tumor. However, there is insufficient evidence to demonstrate the usefulness of FDG-PET/CT for staging and re-staging of GIST, and there is no clear answer as to whether FDG-PET/CT should be used in combination with contrast-enhanced CT or whether it should be replaced by FDG-PET alone. FDG-PET/CT has problems in terms of exposure, cost, availability, and insurance underwriting conditions. Although the benefits slightly outweigh the harms when the balance of benefits and harms is viewed comprehensively, the evidence is not strong. Based on the above, we weakly recommend the use of FDG-PET/CT for staging and re-staging of GIST [27–33].

#### Radiology 4 (CQ): Is additional FDG-PET useful for evaluation of the drug effect on GIST?

- **Recommendation:** We suggest that FDG-PET is additionally performed for evaluation of the drug effect on GIST.
- **Strength of recommendation:** 2 (Weak recommendation)
- **Quality of evidence:** C (Low quality)
- **Consensus rate:** 100%

FDG-PET can capture metabolic changes in tumors. Overseas, the European Organisation for Research and Treatment of Cancer (EORTC) has defined criteria for determining the efficacy of drug therapy using FDG-PET/CT, based on the change in quantitative values, such as SUV (Standardized Uptake Value), compared to baseline [34]. There are also Response Evaluation Criteria in Solid Tumors (RECIST)-compliant criteria called the Positron Emission Tomography Response Criteria In Solid Tumors 1.0 (PERCIST 1.0) [35]. The number of times drug therapy is administered and the intervals between FDG-PET/CT examinations vary depending on the protocol. It is generally recommended that after the start of drug therapy FDG-PET/CT should be performed at least 10 days apart, in consideration of the flare phenomenon (a phenomenon in which FDG accumulation increases after the start of drug therapy due to increased activity of immune cells, etc., regardless of the response) [35]. In Japan, although FDG-PET/CT was approved in April 2010 for the staging and diagnosis of metastasis or recurrence in patients with GIST, it has not yet been approved for determining the efficacy of drug therapy.

Few comparative studies have examined the effectiveness of adding FDG-PET/CT to CT as a routine examination to determine the efficacy of drug therapy for GIST. Therefore, although CT is commonly used in Japan during follow-up to assess the effectiveness of drug therapy for GIST, there is insufficient evidence that it improves patients' prognosis and quality of life.

The systematic review results showed that the integrated value was 5.657 (95% CI 2.634–12.15, *p* < 0.001), indicating that the addition of FDG-PET/CT has a significantly higher diagnostic odds ratio and helps determine the efficacy of pharmacological therapy for GIST [36–39]. One report evaluated time to treatment failure (TTF) as an endpoint in all GIST patients treated with 400 mg/day or 800 mg/day of imatinib, although the type of PET/CT used in the 18 case count studies was coincidence PET, which is currently not widely used in general clinical practice. The common point among all reports was that metabolic changes assessed by PET were better predictors of treatment response and prognosis than size changes evaluated by CT. However, CT and FDG-PET/CT are radiation exposure examinations, although to a lesser extent. Therefore, to carry out both tests every time to assess efficacy in all cases of GIST is not acceptable. No reports investigating the risk–benefit relationship between CT and FDG-PET/CT were found in the literature we searched. This is an issue to be addressed in the future.

Although FDG-PET was approved to receive insurance coverage in April 2010 for GIST staging and diagnosis of metastasis or recurrence, it has not yet been approved for evaluating response to drug therapy. Since it is clear from this systematic qualitative review that FDG-PET/CT can more accurately determine efficacy by adding FDG-PET/CT, and since this test is already in use overseas, we fully expect that it will be covered by insurance in the future, and we have decided to make a recommendation for this CQ.

Based on the above background, CT is used in Japan to determine the efficacy of drug therapy for GIST. Still, it is desirable to perform FDG-PET/CT, especially in cases with a high risk of peritoneal dissemination because it is more accurate when added to CT [36–39].

## Pathological diagnosis part

### Overview of the pathological diagnosis part

#### Pathological diagnosis of GIST

##### Histology and immunohistochemistry

GIST is histologically composed of spindle cells or epithelioid cells. In the spindle cell type, the tumor cells proliferate in a fascicular or whorl pattern [40–42]. Skeinoid fibers, which are characterized by deposits of eosinophilic materials, are frequently present in small intestinal GISTs. In the epithelioid cell type, tumor cells have rounded nuclei and proliferate in a diffuse, sheet-like pattern, often accompanied by myxoid stroma. In both types, various degrees of hemorrhage and/or necrosis can be seen.

Approximately 95% of GISTs are immunohistochemically positive for KIT, and CD34, alpha-smooth muscle actin, and S-100 protein are positive in 60–80%, 20–40%, and 5% of cases, respectively [40–42]. Up to 5% of GISTs are negative for KIT by immunohistochemistry (IHC), and most of them have a gastric location, epithelioid cell morphology, and *PDGFRA* gene mutation. DOG1 is positive in the vast majority (> 95%) of GISTs, irrespective of KIT positivity.

##### Differential diagnosis

The differential diagnoses of GIST include spindle cell tumors such as leiomyoma, leiomyosarcoma, schwannoma, desmoid-type fibromatosis, inflammatory myofibroblastic tumor (IMT), and solitary fibrous tumor (SFT). Epithelioid cell type GIST should be distinguished from poorly differentiated carcinoma, neuroendocrine tumor, malignant melanoma, glomus tumor, and PEComa [40].

#### Recurrence risk classification of GIST

##### Classification systems

It is not easy to draw a sharp line between “benign” and “malignant” in the case of localized GIST. Instead, recurrence risk classification based on a combination of tumor size and mitotic count is used, and GISTs are classified into very low, low, intermediate (moderate), and high risk. Initially, Fletcher/NIH classification was introduced and became widely accepted (Table 5) [41]. Subsequently, the Miettinen/AFIP classification based on tumor size, mitotic counts, and tumor site was proposed to predict the risk of recurrence because the biological behavior of GIST varies depending on tumor site (Table 6) [42]. Furthermore, since tumor rupture is a strong indicator of local recurrence and/or peritoneal metastasis of GIST, the modified Fletcher/Joensuu classification based on tumor rupture in addition to the above-mentioned three factors is reported to be useful to identify high-risk groups (Table 7) [43, 44].

**Table 5:** Fletcher/NIH consensus classification [41]

Recurrence risk	Tumor size (cm)	Mitotic count (/50 HPFs)
Very low	< 2	< 5
Low	2–5	< 5
Intermediate	< 5	6–10
	5–10	< 5
High	> 5	> 5
	> 10	Any
	Any	> 10

HPFs, high-power-fields

**Table 6:** Miettinen/AFIP classification [42]

Tumor parameters		Recurrence risk classification (%)^a^			
Tumor size (cm)	Mitotic count (/50 HPFs)	Stomach	Small intestine	Duodenum	Rectum
< 2	< 5	None (0)	None (0)	None (0)	None (0)
> 2 to ≤ 5	< 5	Very low (1.9)	Low (4.3)	Low (8.3)	Low (8.5)
> 5 to ≤ 10	< 5	Low (3.6)	Moderate (24)	High (34)^c^	High (57)^c^
> 10	< 5	Moderate (12)	High (52)		
< 2	> 5	None (0)^b^	High (50)^b^	No data^d^	High (54)
> 2 to ≤ 5	> 5	Moderate (16)	High (73)	High (50)	High (52)
> 5 to ≤ 10	> 5	High (55)	High (85)	High (86)^c^	High (71)^c^
> 10	> 5	High (86)	High (90)		

^a^% of patients with recurrence/metastasis based on long-term follow-up studies on large series of GISTs

^b^Tumor categories with very small numbers of cases

^c^Two (upper and lower) categories are combined because of small number of cases

^d^There were no identical cases

**Table 7:** Modified Fletcher/Joensuu classification [43, 44]

Tumor parameters		Recurrence risk classification	
Tumor size (cm)	Mitotic counts (/50 HPFs)^a^	Stomach	Other sites
< 2	< 5	Very low	Very low
> 2 to ≤ 5	< 5	Low	Low
> 5 to ≤ 10	< 5	Intermediate	High
< 2	> 5 to ≤ 10	Intermediate	High
> 2 to ≤ 5	> 5 to ≤ 10	Intermediate	High
> 5 to ≤ 10	> 5 to ≤ 10	High	High
Tumor size > 10 cm (any mitotic counts)		High	High
Mitotic counts > 10/50 HPFs (any tumor size)		High	High
Presence of tumor rupture (any mitotic counts and/or tumor size)		High	High

Initially proposed in Ref. [43] and subsequently modified in Ref. [44]. These guidelines are based on Ref. [44] with modifications

^a^“50 HPFs” is not clearly defined in Refs. [43, 44]. These guidelines define "50 HPFs" as being identical to 5 mm^2^

The assigned risk category can differ depending on the adopted classification system even in an individual tumor. In addition, if the tumor size or mitotic count has a borderline score (e.g. around 5 cm size or 5/5 mm^2^ mitoses), the risk category can be up- or down-graded depending on the pathological assessment. The contour maps are created based on tumor size, mitotic counts, site, and rupture, and show the non-linear areas of estimated recurrence rate. The maps are thought to help physicians when explaining the recurrence risk to patients [45].

##### How to count the mitotic figures

Pathologists should pay attention to the field number (diameter of the eyepiece lens of the microscope) when counting the mitotic figures. In Miettinen’s risk classification, mitotic counts are defined as those across 50 field areas (50-high-power-fields; HPFs) with a combination of field number 14 eyepiece lens and magnification × 40 objective lens, and the 50 HPFs areas are almost equal to 5 mm^2^. The 21 HPFs with a combination of field number 22 eyepiece lens (common in routine diagnosis at present) and magnification × 40 objective lens are identical to 5 mm^2^. Thus, “50 HPFs” with a field number 22 eyepiece lens and magnification × 40 objective lens is much larger than 5 mm^2^; if mitoses were counted across “50 HPFs” with this microscopic condition, the mitotic activity would be overestimated. In order to avoid the discrepancy among observers or microscopes (for standardization), these guidelines recommend evaluating mitotic counts per 5 mm^2^ [46]. Table 8 shows the field numbers of each eyepiece lens and how to convert to 5 mm^2^. For example, mitotic figures are counted across 21 HPFs with combination of field number 22 eyepiece lens and magnification × 40 objective lens. Alternatively, the number of mitotic counts in 11.9 mm^2^ (= “50 HPFs” with combination of field number 22 eyepiece lens and magnification × 40 objective lens) × 0.42 equals that in 5 mm^2^.

**Table 8:** Relationship between field number/diameter of eyepiece lens and field areas

Field number	Diameter (mm)	Field area (mm^2^)	Upper: number of fields per 5 mm^2^
			Lower: field areas of a total of 50 HPFs, and how to convert to 5 mm^2^
14	0.35	0.096	52.1
			50 HPFs = 4.8 mm^2^, × 1.04
16	0.40	0.126	39.7
			50 HPFs = 6.3 mm^2^, × 0.79
18	0.45	0.159	31.4
			50 HPFs = 7.95 mm^2^,, × 0.63
20	0.50	0.196	25.5
			50 HPFs = 9.8 mm^2^,, × 0.51
22	0.55	0.238	21
			50 HPFs = 11.9 mm^2^, × 0.42
24	0.60	0.283	17.7
			50 HPFs = 14.15 mm^2^, × 0.35
26	0.65	0.332	15.1
			50 HPFs = 16.6 mm^2^, × 0.30

##### Gene alterations in GIST

The most common driver of gene alteration in GIST is the c-*kit* mutation; in particular, 70–80% of GISTs harbor the c-*kit* exon 11 mutation [47–49]. GISTs with the c-*kit* exon 11 mutation often show spindle cell morphology and a wide spectrum of biological behavior; however, GISTs with deletions involving codons 557 and 558 tend to be associated with a higher risk of recurrence when adjuvant therapy is not introduced after surgical resection [49]. Approximately 5–10% of GISTs have a c-*kit* mutation at exon 9 and those cases are usually spindle cell tumors of the small intestine, and tend to have a higher risk of recurrence. Mutations in c-*kit* exon 8, 13, and 17 are very rare.

The *PDGFRA* mutation is present in about 10% of GISTs, and the mutation is most commonly located in exon 18, followed by exons 12 and 14 [47, 50]. Most *PDGFRA*-mutant GISTs are epithelioid cell type tumors of the stomach and are biologically indolent. Up to 10% of GISTs are so-called wild-type GISTs lacking both c-*kit* and *PDGFRA* mutations. Most wild-type GISTs are succinate dehydrogenase (SDH)-deficient GIST or NF1-related GIST, whereas *BRAF*-mutant or *RAS*-mutant GIST is very rare [47].

Here, it should be noted that certain subtypes such as *PDGFRA* exon 18 D842V-mutant GIST, SDH-deficient GIST, NF1-related GIST, and *BRAF*-mutated GIST are usually resistant to imatinib.

### Questions

#### Pathology 1 (BQ): Are histological diagnosis by hematoxylin–eosin (HE) staining and immunohistochemistry for KIT useful for differential diagnosis of GIST?

- **Recommendation:** We recommend that histological diagnosis by HE staining and immunohistochemistry for KIT are carried out for differential diagnosis of GIST.
- **Strength of Recommendation:** 1 (Strong recommendation)
- **Quality of evidence:** C (Low quality)
- **Consensus rate:** 100%

GISTs show spindle cell or epithelioid cell morphology on HE. Some cases show mixed spindle cell and epithelioid cell patterns. Approximately 95% of GISTs are immunohistochemically positive for KIT. When the histological appearance is consistent with that of typical GIST and KIT is immunopositive, the diagnosis of GIST is straightforward [51–53]. The differential diagnoses of GIST include spindle cell tumors such as leiomyoma, leiomyosarcoma, schwannoma, desmoid-type fibromatosis, inflammatory myofibroblastic tumor (IMT), and solitary fibrous tumor (SFT). Epithelioid cell type GIST should be distinguished from poorly differentiated carcinoma, neuroendocrine tumor, malignant melanoma, and glomus tumor. These tumors are usually negative for KIT; the finding is useful for differential diagnosis, although pathologists should pay attention to the fact that neuroendocrine tumor and malignant melanoma can express the KIT protein.

Anti-KIT antibodies available for routine diagnosis include rabbit monoclonal antibody and rabbit polyclonal antibody [54, 55]. It should be noted that KIT polyclonal antibody can show non-specific, false-positive staining when pretreatment is inappropriate [54]. In addition, poor fixation of the tissue specimen can lead to a false-negative result of KIT immunostaining. Quality control of IHC should be conducted in each laboratory.

Since most of the references concerning this BQ are retrospective analyses of case series, the evidence level is low. However, through discussion among GIST experts, it has been confirmed that the utility has been widely accepted in practical diagnosis. Thus, we think that the strength of recommendation is “strong.”

#### Pathology 2 (BQ): Is immunohistochemistry for markers other than KIT useful for differential diagnosis of GIST?

- **Recommendation:** We recommend that immunohistochemistry for markers other than KIT is carried out for differential diagnosis of GIST.
- **Strength of Recommendation:** 1 (Strong recommendation)
- **Quality of evidence:** C (Low quality)
- **Consensus rate:** 88.2%

DOG1 is a highly sensitive and specific marker for GIST. The vast majority (~ 95%) of GISTs are immunopositive for DOG1. Up to 5% of GISTs are immunonegative for KIT, but they are basically positive for DOG1 [56]. SDH-deficient GISTs show loss of SDHB by IHC [57]. Non-GIST tumors show a characteristic expression pattern of markers other than KIT and DOG1 [40]. Desmin is usually negative or very focally expressed in GIST, whereas leiomyomas show diffuse and strong positivity for desmin. In leiomyomas, the neoplastic cells are negative for KIT, but KIT-positive mast cells and interstitial cells of Cajal (ICC) are often intermingled; this finding should not be confused with GIST. CD34 is positive for 60–80% of GISTs. Solitary fibrous tumor (SFT) is also positive for CD34, and characteristically shows nuclear expression of STAT6. Nuclear expression of beta-catenin for desmoid fibromatosis and ALK expression for inflammatory myofibroblastic tumor (IMT) are also useful to distinguish GIST. S-100 protein is usually negative or can be very focally positive in GIST, but GIST never exhibits diffuse S-100 positivity like that in schwannoma and malignant melanoma. If making a definite diagnosis is difficult even after immunohistochemical staining, consultation with an expert pathologist should be considered.

Since most of the references concerning this BQ are retrospective analyses of case series, the evidence level is low. However, through discussion among GIST experts, it has been confirmed that the utility has been widely accepted in practical diagnosis. Thus, we think that the strength of recommendation is “strong.”

#### Pathology 3 (BQ): Is gene analysis useful for diagnosis of KIT-negative or KIT-weak GIST?

- **Recommendation:** We suggest that gene analysis is carried out for diagnosis of KIT-negative or KIT-weak GIST.
- **Strength of Recommendation:** 2 (Weak recommendation)
- **Quality of evidence:** C (Low quality)
- **Consensus rate:** 100%

About 5% of GISTs are immunohistochemically negative for KIT. In particular, KIT is often negative or weakly positive in *PDGFRA*-mutant GISTs [58, 59]. Poor fixation of the tissue specimen can also lead to weak immunoreactivity for KIT. As mentioned above, the histological diagnosis of such GIST is usually achieved through a combination of DOG1 and other ancillary markers. Additional molecular testing for c-*kit* and *PDGFRA* can lead to more confident diagnosis. Dedifferentiated GIST is an extremely rare tumor which is immunohistochemically negative for KIT even though a c-*kit* mutation is present [60].

Since most of the references concerning this BQ are retrospective analyses of case series, the evidence level is low. Although there is a consensus about the diagnostic utility of mutational analysis based on discussion among GIST experts, the detected genotype should not markedly change the therapeutic strategy. Thus, we think that the strength of recommendation is “low.”

#### Pathology 4 (BQ): Are frequency and malignant potential different in GIST depending on the primary site?

- **Recommendation:** Frequency and malignant potential are different in GIST depending on the primary site.
- **Strength of Recommendation: –**
- **Quality of evidence:** –
- **Consensus rate:** 100%

It is important to define the term “malignancy” because the prognosis of GIST patients is quite different before and after the introduction of molecular-targeted drugs such as imatinib. Before the introduction of molecular-targeted therapy, it was thought that the prognoses of patients were closely correlated with the recurrence or metastasis of GIST. However, after the introduction of molecular-targeted therapy, the prognosis of GIST patients has dramatically improved and tumor recurrence or metastasis has not always remained consistent with a poor prognosis. Therefore, in these guidelines, we define the malignancy of GIST as the risk of tumor metastasis and recurrence.

Until now, there have been several proposals for classifying the risk of GIST recurrence. Their usefulness has been proved in subsequent comparative observational studies. GISTs develop most often in the stomach (50–70%), followed by duodenum and small intestine (20–30%), and colon (5–10%, most in the rectum), and rarely in the esophagus, mesentery, or omentum. [41–43, 61–63].

Because GISTs developing in sites other than the stomach are reported to have a higher risk of recurrence than gastric GISTs, gastric, duodenal, small intestinal, and rectal GISTs in the Miettinen classification, and gastric and non-gastric GISTs in the modified Fletcher classification are each individually assessed for recurrence risk. [41, 42].

Since most of the references concerning this BQ are retrospective case series, it is difficult to assign the evidence level. However, through discussion among GIST experts, a consensus has been reached about this BQ. This is not a BQ related to usefulness, and determining the strength of recommendation is, therefore, not applicable.

#### Pathology 5 (BQ): Are risk classifications for recurrence useful for evaluation of the biological behavior of GIST?

- **Recommendation:** We recommend that risk classifications for recurrence in GIST are carried out for evaluation of the biological behavior of GIST.
- **Strength of Recommendation:** 1 (Strong recommendation)
- **Quality of evidence:** C (Low quality)
- **Consensus rate:** 88.2%

Because all GIST risk classifications listed in these guidelines have been proved to efficiently extract GISTs at high risk for recurrence [41–43], it is important to assess surgically resected GISTs based on any risk classification for recurrence prediction or indication for adjuvant imatinib therapy.

Although some GISTs might be classified in a different risk category in each classification, basically all classifications extract GISTs at high risk for recurrence so efficiently that those differences are acceptable. The risk assessment in Tables 1, 2 and 3 is a discrete classification; i.e., tumor size or borderline mitotic counts decisively influence the GIST risk category, whereas contour maps continuously assess the risk of GIST recurrence, making them useful when explaining individual probabilities of recurrence [45].

However, in cases of SDH-deficient GISTs, distant metastases have been reported regardless of the risk categories of conventional classifications, so prediction of metastasis might be difficult in SDH-deficient GISTs [64].

Since most of the references concerning this BQ are retrospective analyses of case series, the evidence level is low. However, through discussion among GIST experts, a consensus has been reached about the utility of risk classification in practical diagnosis. Thus, we think that the strength of recommendation is “strong.”

#### Pathology 6 (BQ): Is taking a biopsy specimen useful in the evaluation of malignant potential (recurrence risk) in GIST?

- **Recommendation:** We suggest that taking a biopsy specimen is not useful in the evaluation of malignant potential (recurrence risk) in GIST.
- **Strength of Recommendation:** 2 (Weak recommendation)
- **Quality of evidence:** C (Low quality)
- **Consensus rate:** 87.5%

In general, it is difficult to obtain sufficient tissue samples of SMTs via a conventional endoscopic biopsy, so it is not easy to histologically diagnose those SMTs as GISTs. However, for cases where sufficient submucosal samples can be obtained by boring biopsy or EUS-FNA biopsy and appropriate IHC is performed, it may be possible to make a histological diagnosis of GISTs [6, 65, 66].

However, accurate risk grading using biopsy specimens is thought to be difficult in most cases because it is difficult to obtain enough tissue for mitotic counting and because mitotic counts are often heterogeneous within the same tumor. A tissue sample of at least 5 mm^2^ is needed to determine the mitotic count for risk grade classification. It is reasonable to assume that some GISTs showing very high mitotic activity to be high risk.

Although IHC using anti Ki-67 antibodies has been used for risk grading of biopsy samples in some reports [6, 66], this method has some pitfalls. Lymphocytic infiltration is frequently observed in GISTs, so the Ki-67 labeling index might be overestimated in such cases; moreover, field biases of Ki-67-positive cells are also seen in many GISTs. Thus, risk grading using small biopsy specimens is not recommended in these guidelines.

Since there are small numbers of retrospective analyses concerning this BQ, the evidence level is low. The statement of this BQ is based on those references and the experience of GIST experts. Thus, we think that the strength of recommendation is “low.”

#### Pathology 7 (BQ): Are there any correlations between KIT immunohistochemisry results and c-kit mutational status?

- **Recommendation:** There are no apparent correlations between KIT immunohistochemisry results and c-*kit* mutational status.
- **Strength of Recommendation: –**
- **Quality of evidence: –**
- **Consensus rate:** 100%

Immunoreactivity for KIT in GIST includes cytoplasmic, membranous or combined cytoplasmic, and membranous patterns. Golgi pattern is also occasionally seen. The KIT expression pattern is not associated with genotype or exon site of the c-*kit* mutation [67]. *PDGFRA*-mutant GISTs usually show negative or weak expression of KIT, but some cases are immunopositive for KIT. Rare variants of c-*kit*-wild GISTs such as SDH-deficient, NF-related, and BRAF-mutated type are usually positive for KIT by IHC [68–70]. Again, there are no definite relationships between KIT expression pattern and the presence of the c-*kit* mutation.

Since most of the references concerning this BQ are retrospective case series, it is difficult to assign the evidence level. However, through discussion among GIST experts, a consensus has been reached about this BQ. This is not a BQ related to usefulness, and determining the strength of recommendation is therefore not applicable.

#### Pathology 8 (BQ): Is mutational analysis useful for evaluation of primary imatinib-resistant GIST?

- **Recommendation:** We suggest that mutational analysis is carried out for evaluation of primary imatinib-resistant GIST.
- **Strength of Recommendation:** 2 (Weak recommendation)
- **Quality of evidence:** D (Very low quality)
- **Consensus rate:** 94.1%

Some patients diagnosed as having unresectable and/or recurrent GISTs show primary imatinib resistance. Those GISTs are basically considered to have primary imatinib-resistant gene alterations [71]. Most of GISTs with the c-*kit* mutation are imatinib-sensitive, while above half of the GISTs with *PDGFRA* mutation are primarily imatinib resistant. In particular, imatinib is considered be ineffective for cases where there is a D842V mutation in *PDGFRA* exon 18. Moreover, most GISTs without c-*kit* and *PDGFRA* mutations as described in Pathology 9 (BQ) usually show primary resistance to imatinib although those cases are rare. Since tyrosine kinase inhibitors (TKIs) such as imatinib, sunitinib, and regorafenib are now used for unresectable and/or recurrent GISTs and since sunitinib and regorafenib are administered in that order in cases of primary imatinib resistance, it is not necessary to clarify the mutation status in GISTs. However, we could change the drug from imatinib to sunitinib at an earlier time in imatinib-resistant/intolerant GISTs proved to have the exon 9 c-*kit* mutation by gene analyses since sunitinib is expected to be more effective in treating such GISTs, which often show primary imatinib resistance. Furthermore, in primarily imatinib-resistant tumors diagnosed as GISTs, there is a possibility that the diagnosis of GIST is not accurate. In those cases, we have to check the diagnosis. In summary, the usefulness of mutational analysis in primary imatinib-resistant GISTs is limited.

Although mutational analyses in GISTs are considered to be clinically relevant based on discussion among GIST experts, most of the references concerning this BQ are retrospective case series and the evidence level is very low. Thus, we think that the strength of recommendation is “weak.”

#### Pathology 9 (BQ): Are there any GISTs caused by gene abnormalities other than c-kit and PDGFRA mutations?

- **Recommendation:** There are GISTs caused by gene abnormalities other than c-*kit* and PDGFRA mutations.
- **Strength of Recommendation: –**
- **Quality of evidence: –**
- **Consensus rate:** 100%

Seventy-five to 85% of GISTs have a c-*kit* mutation while approximately 10% of them have a *PDGFRA* mutation. The other GISTs (approximately 10%) include those associated with NF1 patients (1–2%) [72], those with *SDHs* mutation (2–5%) [73], those with *BRAF* mutations (− 1%) [74], and those with other gene abnormalities (some %). Some cases of GISTs with a *KRAS* mutation have been reported, but most of them are considered to have the mutation in addition to a c-*kit* mutation in secondary lesions resistant to TKIs and in far advanced cases [60]. Although GISTs with the *NTRK* fusion gene have been reported, a recent report claimed that such gastrointestinal mesenchymal tumors are not true GISTs [75]. Thus, it is not clear whether GISTs with the *NTRK* fusion gene are present or not.

In GISTs with neither a c-*kit* mutation nor *PDGFRA* mutation, imatinib is usually ineffective. Thus, neoadjuvant and adjuvant imatinib therapies for those GISTs might not be considered. Even in GISTs with a c-*kit* mutation or *PDGFRA* mutation, imatinib might be ineffective in GISTS with some particular types of mutation. The association between gene mutations in GIST and the efficacy of imatinib is describe in Pathology 8 (BQ).

Diagnosis and therapy for GISTs with neither the c-*kit* mutation nor the *PDGFRA* mutation require consultation with experts in this area or should be referred to hospitals specializing in GISTs and/or sarcomas.

Since most of the references concerning this BQ are retrospective case series, the evidence level is low. However, through discussion among GIST experts, a consensus has been reached concerning this BQ as many case series have been reported. This is not a BQ related to usefulness, and determining the strength of recommendation is therefore not applicable.

#### Pathology 10 (BQ): Are there any pathological conditions with multiple GISTs?

- **Recommendation:** There are pathological conditions with multiple GISTs.
- **Strength of Recommendation: –**
- **Quality of evidence: –**
- **Consensus rate:** 100%

Most GISTs, which develop sporadically and singularly, are associated with the somatic c*-kit* or *PDGFRA* mutation, but multiple GISTs, each with a different somatic mutation, rarely develop sporadically. Moreover, there are the following multiple familial or syndromic GISTs [76–78].

- Familial GISTs with inherited germline mutations of the c*-kit* or *PDGFRA* gene
  To date, more than 30 families of inherited GISTs have been reported. Inherited GISTs have c*-kit* or *PDGFRA* mutations just as sporadic GISTs do. In families with germline c*-kit* mutations, multiple GISTs develop in the stomach and small intestine associated with hyperplasia of ICC. On the other hand, in families with germline *PDGFRA* mutations, multiple GISTs develop only in the stomach, and inflammatory fibroid polyps or lipomas also develop in some cases. Separate from families with c*-kit* mutations, hyperplasia of ICC has not been reported in families with *PDGFRA* mutations.
- NF1-associated GISTs
  There have been reports of GISTs developing in some neurofibromatosis type 1 patients. In most cases, multiple GISTs develop in the small intestine and rarely in the stomach. Although dozens or hundreds of GISTs develop in some cases, they must not be mistakenly identified as tumor dissemination. Hyperplasia of ICC is also seen in the myenteric plexus.
- Carney–Stratakis syndrome and Carney triad
  In patients with Carney–Stratakis syndrome, in which the development of GISTs and paragangliomas is an inherited condition, and in Carney triad patients, in which the development of GISTs, paragangliomas, and pulmonary chondromas is not an inherited condition, GISTs lacking expression of SDHB protein develop in the stomach, most of which are multifocal. One of the germline mutations of SDHB, SDHC, or SDHD genes, which encode subunits of the SDH enzyme complex, has been reported in families with Carney–Stratakis syndrome, and hypermethylation of the SDHC promoter region and subsequent decrease of gene expression have been reported in Carney triad tumors. Hyperplasia of ICC has not been reported in these cases.

Since most of the references concerning this BQ are retrospective case series, the evidence level is low. However, through discussion among GIST experts, a consensus has been reached concerning this BQ as many case series have been reported. This is not a BQ related to usefulness, and determining the strength of recommendation is therefore not applicable.

## Surgical management part

### Overview of the surgical management part

Surgery is the primary treatment for resectable localized GIST without metastasis, however, treatment strategies and surgical procedures may vary depending on the size and anatomical location of the tumor. Although administration of imatinib is the first choice as primary treatment for unresectable metastatic or recurrent GIST, imatinib-resistant GIST is often difficult to treat after second-line treatment. Therefore, the indication for surgery in metastatic or recurrent GIST treated with TKIs needs to be discussed.

#### Surgery for primary GIST

##### Treatment strategies for resectable localized submucosal tumor (SMT)

In Japan, relatively small gastric SMTs are often detected on endoscopic screening for upper gastrointestinal tract diseases. Given this situation, Algorithm 4 “treatment strategies for resectable localized SMTs” (Fig. 4) was established mainly for gastric SMT. Surgery 1 (CQ) and 2 (CQ) were established because treatment strategies may be controversial for pathologically diagnosed gastric GISTs of less than 2 cm, or SMTs sized 2–5 cm. Although no previous reports analyzed the prognosis of gastric GISTs less than 2 cm, nor showed the usefulness of surgery for such small GISTs, surgery is suggested for pathologically diagnosed GISTs or small SMTs with malignant features strongly suggestive of GIST or other malignant tumors, based on the results of retrospective cohort studies for resected GIST [45, 62], and considering the safety of surgery and the high complete resection rates [79] [Algorithm 4 (Fig. 4), Surgery 1 (CQ)]. Surgery is also recommended for SMTs sized 2–5 cm and strongly suspected of being GISTs or other malignant tumors [Algorithm 4 (Fig. 4), Surgery 2 (CQ)].

##### Surgery, neoadjuvant therapy and adjuvant therapy for localized GIST

Surgery 3 (CQ) was established, because the indication of laparoscopic surgery remained controversial for GISTs 5 cm or larger and for SMTs sized 2–5 cm and strongly suspected of being GISTs or other malignant tumors, while it is usually performed for relatively small GISTs less than 5 cm. Laparoscopic surgery may be indicated for GISTs 5 cm or larger, based on the results of meta-analyses that compared the outcomes of open surgery and laparoscopic surgery for GISTs 5 cm or larger [80–82]. However, laparoscopic surgery is not necessarily recommended for GISTs larger than 8 cm because there is no sufficient evidence to show the superiority of laparoscopic surgery to open surgery for such large GISTs [Algorithm 4 (Fig. 4), Surgery 3 (CQ)]. Although organ function-preserving surgery is recommended for GISTs requiring surgical resection [Surgery 4 (BQ)], it is more important to prevent tumor rupture and to achieve complete resection. Therefore, the usefulness of neoadjuvant therapy for GISTs 10 cm or larger was investigated in a multi-institutional phase II study conducted in Japan and Korea, which have a high R0 resection rate [83]. Neoadjuvant imatinib therapy is suggested for large GISTs 10 cm or larger and for GISTs for which incomplete resection or intraoperative tumor rupture is suspected [Algorithm 5 (Fig. 5), Surgery 5 (CQ)]. In cases of preoperative or intraoperative rupture, adjuvant imatinib therapy is recommended after surgery [Algorithm 6 (Fig. 6), Surgery 6 (CQ)].

#### Surgery for metastatic or recurrent GIST

##### Surgery as initial treatment

Because positive effects of hepatectomy on survival were shown in colorectal cancer liver metastasis, and the maximal tumor diameter at the start of imatinib therapy for unresectable metastatic GIST correlated with the progression-free survival (PFS) [84], the usefulness of surgery alone or cytoreductive surgery followed by imatinib therapy were investigated in some prospective cohort studies and retrospective case series studies [85–91]. However, no studies showed evidence for the prognostic impact of surgery alone or surgery followed by imatinib therapy. In addition, a small study showed a positive correlation between the duration of imatinib administration and overall survival (OS) [90]. Taken together, the principal treatment strategy for metastatic or recurrent GIST is considered to be imatinib administration [Algorithm 6, 7 (Figs. 6, 7), Surgery 8 (CQ)].

##### Surgery for GIST treated with TKIs

The usefulness of surgery for GIST responding to or resistant to imatinib was investigated in a small randomized controlled trial (RCT) and some retrospective observation studies [92–94]. Because of the small number of enrolled patients and the latent bias in these studies, there is insufficient evidence to show the usefulness of surgery for GIST treated with TKIs. Surgery for GIST treated with TKIs is regarded as a challenging treatment strategy that should only be performed in specialized hospitals highly experienced in the treatment of GIST or sarcomas [Algorithm 7, 8 (Figs. 7, 8), Surgery 9 (CQ), 10 (CQ)].

### Questions

#### Surgery 1 (CQ): Is surgery recommended for GISTs less than 2 cm?

- **Recommendation:** We suggest that surgery is carried out for GISTs less than 2 cm.
- **Strength of Recommendation:** 2 (Weak recommendation)
- **Quality of evidence:** D (Very low quality)
- **Consensus rate:** 91.7%

The previous edition of the Japanese clinical practice guidelines for GIST recommends surgical resection for gastric GISTs less than 2 cm [96]. The NCCN clinical practice guidelines recommend resection for gastric GISTs less than 2 cm with “high-risk features” and, otherwise, advise regular follow-up with EUS for these GISTs without “high-risk features.” All guidelines recommend surgery for non-gastric GISTs even when they are less than 2 cm [46, 79, 95–97].

There are, however, no prospective cohort studies for gastric GISTs less than 2 cm with endpoints of OS and/or recurrence free survival (RFS) or with control groups that did not undergo resection. An epidemiological study using the SEER (Surveillance Epidemiology and End Results) database, a regional cancer registry in the United States, in which all registered GISTs less than 2 cm have been analyzed and gastric GISTs account for 62% of cases, shows that the 5-year disease-specific mortality rate is 12.9% for patients with GISTs less than 2 cm alone, and it increases to 31.4% or 36.5% when there is lymph node or distant metastases, respectively. In this study, 5-year disease-specific mortality rate with resection was 17.5% and that without resection was 39.8% [98]. However, it should be kept in mind that the SEER data do not include GISTs which do not undergo surgery and subsequent pathological diagnosis.

Several retrospective cohort studies showed that the 10-year postoperative RFS rates in patients with GISTs less than 2 cm exhibited a slight decrease of a few percent, indicating recurrence even after complete resection of small GISTs [45, 62]. Some case reports or case series also included gastric GISTs less than 2 cm with distant metastasis [99, 100]. It may be considered that wedge resection, which is applicable for most small gastric GISTs, is safe and feasible, and the R0 resection rate is considered high.

In summary, although there are neither controlled studies evaluating the prognosis of patients with gastric GISTs less than 2 cm nor reports showing the effectiveness of surgery for these small GISTs, the expert consensus suggests that surgery is carried out for small gastric GISTs less than 2 cm.

#### Surgery 2 (CQ): Is surgery recommended for submucosal tumors (SMTs) between 2 and 5 cm?

- **Recommendation:** We recommend that surgery is carried out for SMTs sized 2–5 cm which are diagnosed as GISTs or strongly suspected of being malignant tumors including GISTs.
- **Strength of Recommendation:** 1 (Strong recommendation)
- **Quality of evidence:** C (Low quality)
- **Consensus rate:** 100%

There has been no study that investigated whether surgical resection of undiagnosed SMTs between 2 and 5 cm is beneficial and/or may improve the prognosis (including OS, RFS, etc.) of patients with these tumors. All original reports identified in the secondary screening are retrospective cohort studies, four of which targeted SMTs, and are feasibility studies to examine the safety of surgical procedures. Eight studies focused on surgical resection of GISTs, and none of the studies have appropriate control groups, such as patients without surgery.

Several reports have evaluated the relationship between tumor size of gastric GISTs and recurrence after surgery [45, 62, 101]. A report from Japan has compared RFS of patients with GISTs between 2 and 5 cm, those between 5.1 and 10 cm, and those greater than 10.1 cm to that of patients with GISTs less than 2 cm and has found that the former three groups have poorer prognosis with a hazard ratio (HR) of 5.91 (95% CI 0.79–44.01, *p* = 0.0829), 28.25 (95% CI 3.82–208.83, *p* < 0.0001), 51.75 (95% CI 6.80–394.07, *p* < 0.0001) [62].

Harms associated with surgical resection (adverse events, functional impairment, etc.) are infrequent and often mild, if present [79].

The above-mentioned retrospective studies included patients who had undergone surgery due to GISTs or due to SMTs suspected of being malignant tumors, or patients who were considered to require surgery, i.e., because of symptoms. In this connection, clinical findings and presentation of SMTs suggestive of malignant tumors may include tumor ulceration, heterogeneous echo in EUS, irregular margins, and increase in size during follow-up, as significant factors based on a consensus reached by experts, which have been indicated by retrospective observational studies [79, 96].

Taken together, since the targeted tumors analyzed are “thought to require surgical resection” as mentioned above, we recommend that surgery is carried out for SMTs sized 2–5 cm “which are strongly suspected of being GISTs or other malignant tumors.”

#### Surgery 3 (CQ): Is laparoscopic surgery recommended for submucosal tumors (SMTs) 5 cm or larger?

- **Recommendation:** We suggest that laparoscopic surgery is carried out for SMTs 5 cm or larger.
- **Strength of Recommendation:** 2 (Weak recommendation)
- **Quality of evidence:** D (Very low quality)
- **Consensus rate:** 100%

An SMT can usually be completely removed by simple resection without reconstructing the digestive tract, as no regional lymph node dissection is required. A simple, local resection is compatible with a minimally invasive approach, and gentle manipulation to avoid tumor rupture is important for both the open surgery and laparoscopic approaches. There is no evidence supporting a cut-off value for the size for tumors which can be safely resected laparoscopically without injury to the tumor itself.

Three meta-analyses, including two for gastric GISTs and one for GISTs [80–82], have compared the outcomes of open and laparoscopic surgery for SMTs larger than 5 cm. The short-term outcomes of laparoscopic surgery were favorable or equivalent to those of open surgery in terms of intraoperative bleeding, operation time, perioperative complications, and duration of hospitalization. The long-term outcomes, such as disease-free survival (DFS) and OS, were also more favorable for laparoscopic surgery than open surgery. In oncological terms as well, there are no previous reports demonstrating that the surgical approach affects the risk of a microscopic positive margin or tumor rupture although the number of events may be small. Note that our recommendation is based on data from the meta-analyses showing that open surgery was frequently chosen for tumors larger than 8 cm. Laparoscopic surgery is not recommended for GISTs of considerable size as the procedure is not more beneficial than open surgery.

This CQ applies to all SMTs, given that the tumor location may not be ascertainable or the tissue may not be available in clinical practice. However, most of the articles cited in systematic reviews specified the organ and tissue type of the target disease, especially studies dealing only with gastric GIST, and comprised 68.7% (46/67) of the total number. Therefore, the data from these studies were considered extrapolatable to all SMTs, as gastric GIST is the most common type of SMT while evidence for the other types is scant. The influence of the minor differences in surgical procedures, such as the tumor non-exposure technique and concomitant use of an endoscope on clinical outcome remains controversial, and the supporting evidence is insufficient to allow discussion here.

Laparoscopic surgery for SMT is increasing in Japan year by year. Considering the balance of benefits and harms, strength of evidence, patients’ wishes, etc., we advise that laparoscopic surgery should be considered for SMTs larger than 5 cm.

#### Surgery 4 (BQ): Is organ function-preserving surgery recommended for GISTs requiring surgical resection?

- **Recommendation:** We recommend that organ function-preserving surgery is carried out for GISTs requiring surgical resection.
- **Strength of Recommendation:** 1 (Strong recommendation)
- **Quality of evidence:** D (Very low quality)
- **Consensus rate:** 100%

Since no evidence on the effect of systematic lymph node dissection is available, local resection of the primary organ without lymph node dissection is recommended as a standard surgical procedure for primary GIST. Securing an adequate tumor margin and preserving organ function are also strongly recommended except in cases where local resection may impair the motility of the gastrointestinal tract.

There is currently no universally accepted definition of function-preserving gastrointestinal surgery. In this BQ, “function-preserving surgery” is defined as surgery avoiding total organ resection and the removal of sites with specific functions that are difficult or impossible to replicate artificially (cardiac, pyloric, anal, etc.). Esophagectomy, pancreaticoduodenectomy, and rectal amputation are examples of highly invasive procedures that may negatively affect the patient’s postoperative quality of life and should therefore be avoided whenever possible.

**Fig. 9:**
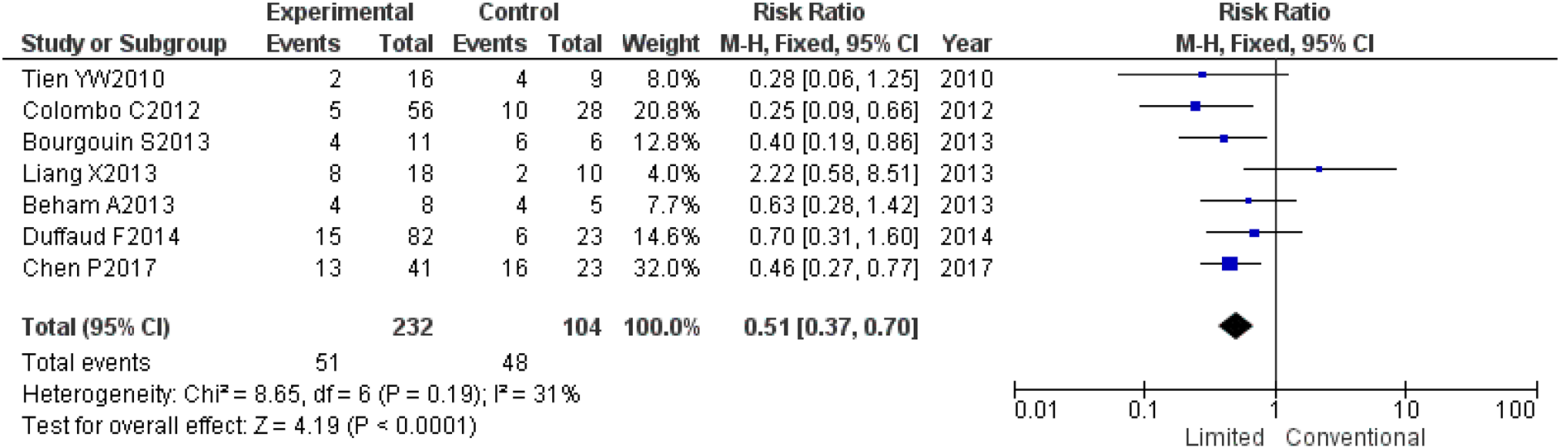
Comparison of duodenal local resection and pancreaticoduodenectomy for duodenal GIST—a meta-analysis of postoperative complications

A systematic review found 15 studies examining function-preserving surgery for GIST, including seven case-controlled studies [102–108] comparing the outcomes between local resection and pancreaticoduodenectomy for duodenal GIST. Our own meta-analysis of these seven studies found that 232 local resections and 104 pancreaticoduodenectomies had been analyzed and that local resection had a risk ratio of 0.51 and was associated with a significantly lower risk of postoperative complications than pancreaticoduodenectomy (95% confidence interval: 0.37–0.70; *p*-value < 0.0001) (Fig. 9).

The risk of tumor rupture and a positive resection margin was examined in a small number of cases and events, but no significant increase in the risk by every type of organ function-preserving surgery was found. Moreover, the indications for function-preserving surgery are likely to change according to the degree of tumor shrinkage in cases treated with neoadjuvant therapy using imatinib. Although no supporting RCT was found and the strength of the evidence was D (very weak), considering the balance of benefits and harms as well as the patient's wishes, organ-function-preserving surgery is strongly recommended for GIST for which surgical resection is indicated.

#### Surgery 5 (CQ): Is neoadjuvant imatinib therapy useful for large GIST and where incomplete resection is suspected?

- **Recommendation:** We suggest that neoadjuvant imatinib therapy is carried out for large GIST 10 cm or larger and for GIST where incomplete resection is suspected.
- **Strength of recommendation:** 2 (Weak recommendation)
- **Quality of evidence:** C (Low quality)
- **Consensus rate:** 100%

No RCT investigating the clinical benefit of neoadjuvant imatinib therapy for resectable GIST has been conducted before. Most of the previous studies retrospectively collected patients who had received neoadjuvant imatinib therapy. That means there is no evidence of the effect on OS or RFS. A prospective phase II study showed that neoadjuvant imatinib therapy could achieve tumor reduction and improve the R0 resection rate. From the results of this phase II study, we weakly recommend that neoadjuvant imatinib therapy should be carried out for GIST ≥ 10 cm and for GIST where incomplete resection is suspected. The indication of neoadjuvant imatinib therapy for non-gastric GIST is still controversial.

We found a prospective single-arm phase II study conducted in Japan and Korea [83]. In this study, 53 patients with gastric GIST ≥ 10 cm received neoadjuvant imatinib therapy for 6–9 months. The primary endpoint was the R0 resection rate. Although the R0 resection rate without neoadjuvant imatinib therapy was expected to be 70% based on previous reports, in this phase II study, a significantly higher R0 resection rate of 91% (95% CI 79–97%; *p* < 0.001) was achieved. The completion rate of neoadjuvant imatinib therapy for ≥ 6 months was 87%, indicating high feasibility.

Another retrospective study showed the median duration of imatinib treatment to the best response as 28 weeks (IQR, 18–37 weeks) and the median tumor reduction rate as 43% (IQR, 31–48%) [109]. Considering the positive results from the phase II study as mentioned above, the optimal duration of neoadjuvant imatinib therapy seems to be 6 months or longer.

The validity of risk classification after neoadjuvant treatment is unclear. The indication of adjuvant imatinib therapy after neoadjuvant treatment is also unknown.

Some retrospective studies included a small number of cases who underwent function-preserving surgery after neoadjuvant imatinib therapy for rectal or duodenal GIST [110–112]. Since all of the studies were retrospective, the benefit of neoadjuvant imatinib therapy on function preservation is unclear.

#### Surgery 6 (CQ): Is adjuvant imatinib therapy useful for GIST with preoperative or intraoperative rupture?

- **Recommendation:** We recommend that adjuvant imatinib therapy is carried out for GIST with preoperative or intraoperative rupture.
- **Strength of recommendation:** 1 (Strong recommendation)
- **Quality of evidence:** B (Moderate quality)
- **Consensus rate:** 100%

Several retrospective studies showed that the prognosis of GIST with tumor rupture was extremely poor. [44, 113, 114]. If tumor rupture is present, the case is classified into the high-risk group according to the risk classification (modified Fletcher classification) [45]. For the high-risk group, adjuvant imatinib therapy is strongly recommended.

In the systematic review, we found no reports focusing solely on patients with tumor rupture. In a SSG XVIII study comparing 1- and 3-year adjuvant imatinib therapy for high-risk GIST, tumor rupture occurred in 35 of the 1-year group and 44 in the 3-year group, and the hazard ratio for recurrence in the 3-year group was 0.47 (95% CI 0.25–0.89) [115].

Besides the SSG XVIII study, three RCTs demonstrated the survival benefit of adjuvant imatinib therapy for intermediate- or high-risk GIST [114, 116, 117]. In the subgroup analyses for GIST with tumor rupture, two RCTs showed a significant survival benefit for adjuvant imatinib therapy, and the remaining one RCT showed a trend toward improving survival after adjuvant imatinib therapy.

The long-term prognosis after adjuvant imatinib therapy for GIST with tumor rupture is still unknown. Research is needed to investigate the optimal duration of adjuvant imatinib therapy for GIST with tumor rupture.

#### Surgery 7 (BQ): Is routine follow-up useful for GIST after complete resection?

- **Recommendation:** We suggest that routine follow-up is carried out for GIST after complete resection.
- **Strength of Recommendation:** 2 (Weak recommendation)
- **Quality of evidence:** D (Very low quality)
- **Consensus rate:** 94.1%

Whether or not regular follow-up improves the survival and quality of life of GIST patients who underwent complete resection (R0-resection) is a clinically relevant issue that should be resolved. Literature review has revealed that there is no determinative interventional study that clarifies the usefulness of regular follow-up in the postoperative management of GIST patients. Nevertheless, many clinical guidelines based on expert consensus have recommended regular follow-up [118, 119].

One retrospective study has addressed the relationship between early diagnosis of GIST recurrence and patient survival [120]. In that study, 233 patients who underwent resection of primary GISTs were followed up and PFS and disease-specific survival after recurrence were analyzed in 94 patients who developed disease recurrence. Multivariate analysis has indicated that asymptomatic cases at recurrence diagnosis and low tumor burden (hepatic metastases with less than four foci, single peritoneal metastasis, or peritoneal metastases whose total major diameter measures 10 cm or less) are statistically significantly favourable prognostic factors. Moreover, a retrospective study based on clinical trial datasets of 818 advanced GIST patients who underwent imatinib therapy revealed that the time to progression (TTP) is significantly longer in patients with a smaller metastasis (the largest diameter being less than 12 cm) than in those with a larger one [84]. These findings suggest that early diagnosis of recurrence may lead to improved patient survival in the management of GISTs. Meanwhile, we also need to acknowledge that the results are considered low-level evidence and may be influenced by lead-time bias.

The optimal interval and the observation period for patient follow-up are still unknown. A retrospective cohort study of 712 Japanese GIST patients has demonstrated that the 5-year disease-free survival rate is approximately 60% for patients with high-risk GISTs, whereas it is approximately 90% for those with intermediate-risk GISTs and 95% or higher for patients with low-risk GISTs [62]. In addition, there is one clinically relevant study in which the timing of recurrence was retrospectively analyzed on the basis of data of a randomized clinical trial that investigated the efficacy of adjuvant imatinib therapy (SSGXVIII/AIO study) [20]. That study revealed that the recurrence hazard significantly increased around 6–12 months after the completion of adjuvant imatinib therapy. Together, these studies suggest that the clinical usefulness of patient follow-up varies depending on the case. The interval and the modality of follow-up should be determined after considering clinical background including recurrence risk, time after surgery, and implementation of adjuvant therapy.

#### Surgery 8 (CQ): Is upfront surgery useful for metastatic GIST?

- **Recommendation:** We suggest that upfront surgery is not carried out for metastatic GIST.
- **Strength of Recommendation:** 2 (Weak recommendation)
- **Quality of evidence:** D (Very low quality)
- **Consensus rate:** 94.1%

It is widely known that metastasectomy offers a significant clinical benefit for improving patient survival as a treatment for liver metastasis of colorectal cancer. This clinical question was raised in a similar clinical context.

Although literature review has revealed several studies on metastasectomy of metastatic GISTs, there are only five studies that adopted metastasectomy as the first-line treatment of metastatic GISTs, consistent with the clinical question. Those studies included one prospective cohort study and four case series studies.

The prospective cohort study was a multi-institutional study conducted in Japan. The key eligible criterion was GIST patients with liver oligometastasis (three or fewer metastatic foci). The patients underwent metastasectomy or imatinib therapy according to their preference, and RFS and PFS of each group were followed. Patients in the metastasectomy group did not receive imatinib therapy prior to the determination of disease relapse after metastasectomy. Although the study was discontinued early due to low patient accrual, the median RFS was as short as 145 days and the 3-year RFS rate was 16.7% in the metastasectomy group. These findings suggest that metastasectomy has little clinical benefit for patients with liver oligometastases of GISTs [85].

In all the four case series studies, many of the enrolled patients underwent imatinib therapy following metastasectomy [87–89, 91]. One study retrospectively compared survivals of patients with and without surgery for resectable metastases of GISTs. In that study, 24 patients who underwent metastasectomy as first-line treatment showed significantly better OS than six patients without metastasectomy [89]. In another study, survivals were retrospectively compared between 23 patients who underwent resection of liver metastases and the following TKI therapy and 98 patients who received TKI therapy alone. That study showed no statistically significant difference in OS between the two groups [91]. In yet another retrospective study that compared survivals of patients who underwent metastasectomy before and after imatinib therapy, no significant difference was noted in PFS and OS between the two groups [88].

Unfortunately, the above-mentioned case series studies were considered low-level evidence studies because they were small in scale and potentially included selection bias related to patient background. The board concluded that there is insufficient solid clinical evidence to recommend upfront surgery for metastatic GISTs, although it remains an open question as to whether a multimodality approach such as metastasectomy plus TKI therapy would improve survival of metastatic GIST patients or not.

#### Surgery 9 (CQ): Is surgery useful for metastatic or recurrent GIST responding to imatinib?

- **Recommendation:** We suggest that surgery is not carried out for metastatic or recurrent GIST responding to imatinib.
- **Strength of Recommendation:** 2 (Weak recommendation)
- **Quality of evidence:** D (Very low quality)
- **Consensus rate:** 94.1%

Although treatment with imatinib is reportedly effective in about 80% of metastatic or recurrent GIST, the results of the B2222 clinical trial showed that secondary resistance to imatinib developed in about half of the patients within 2 years of starting therapy [121]. Therefore, surgical resection may be performed in some cases of metastatic or recurrent GIST to prevent resistance to imatinib, however, the usefulness of such surgery remains unclear. This clinical question was established in response to these background factors.

There is only one RCT corresponding to this CQ, which was terminated early due to low patient accrual. Only 41 patients were enrolled. Nineteen patients who underwent surgery for residual disease after starting imatinib, and 21 patients who received imatinib alone until progression were enrolled in the analysis. There was no statistically significant difference in 2-year PFS between the surgery arm and the imatinib-alone arm (88.4% and 57.7%, *p* = 0.089). Median OS was not reached in the surgery arm and was 49 months in patients in the imatinib-alone arm, which was significantly better in the surgery arm (*p* = 0.024) [92]. There were only three retrospective observation studies, which compared 12, 38, and 42 patients, respectively, who underwent surgery for GIST responding to imatinib with 92, 27, and 144 patients, respectively, who continued imatinib. Of these, one and two studies showed better RFS and OS, respectively, in the surgery group than in the imatinib group [122–124]. However, there was major bias in terms of patient background factors in these observation studies, and the numbers of enrolled patients were too small to establish sufficient evidence.

Although surgery may be useful for certain patients with metastatic or recurrent GIST responding to imatinib, currently there is insufficient evidence demonstrating its usefulness. Because this is a challenging treatment strategy that should only be performed in specialized hospitals highly experienced in the treatment of GIST or sarcomas, we suggest that surgery is not carried out for metastatic or recurrent GIST responding to imatinib based on the consensus reached by GIST specialists.

#### Surgery 10 (CQ): Is surgery useful for metastatic or recurrent GIST resistant to tyrosine kinase inhibitors?

- **Recommendation:** We suggest that surgery is not carried out for metastatic or recurrent GIST resistant to tyrosine kinase inhibitors.
- **Strength of Recommendation:** 2 (Weak recommendation)
- **Quality of evidence:** D (Very low quality)
- **Consensus rate:** 100%

Metastatic or recurrent GIST cannot be cured with TKIs alone, and the treatment often becomes difficult due to acquired resistance to TKIs. Therefore, surgery may be performed in some cases with the intention of achieving R0 resection of imatinib-resistant lesions. However, the usefulness of such surgery remains unclear. This clinical question was established in response to these background factors.

There have only been a few retrospective observation studies but no RCTs corresponding to this CQ. One study compared 38 metastatic or recurrent GIST patients who underwent surgery for partial resistance to imatinib with 19 patients who did not undergo surgery. PFS and OS were significantly better in the surgery group [125]. There were 4 and 2 studies on the long-term outcomes after surgery for metastatic or recurrent GIST with partial resistance and systemic resistance to imatinib, respectively. Postoperative PFS and OS were better in the partial resistance group than in the systemic resistance group [93, 126–128]. There was only one study that compared patients with and without surgery for sunitinib-resistant GIST. PFS and OS were better in 26 patients who underwent surgery than in 43 patients who did not undergo surgery [129]. Two studies reported long-term outcomes after surgery for sunitinib-resistant GIST, which showed no significant correlation between the treatment effect of sunitinib at surgery and PFS or OS [93, 130]. There was huge bias in terms of patient background in these observation studies, and the numbers of enrolled patients were too small to establish sufficient evidence.

Although surgery may be useful for certain patients with metastatic or recurrent GIST resistant to TKIs, currently there is insufficient evidence to show its usefulness. Because this is a challenging treatment strategy that should only be performed in specialized hospitals highly experienced in the treatment of GIST or sarcomas, we suggest that surgery is not carried out for metastatic or recurrent GIST resistant to TKIs based on a consensus reached by GIST specialists.

## Medical management part

### Overview of the medical management part

#### Treatment for metastatic, recurrent, or unresectable GIST

##### Medical treatment of GIST (first-line treatment)

Medical treatment is the first choice if surgery is not indicated due to metastasis or local advancement. When the diagnosis of GIST [Algorithms 1, 2, 3 (Figs. 1, 2, 3)] is histologically confirmed and major organ functions are preserved, imatinib 400 mg/day should be given once a day after food [Medicine 1 (CQ)]. During imatinib treatment, follow-up by regular interviews, blood examination, and radiological imaging [Radiology 4 (CQ)] should be performed as with other cancers. Imatinib treatment should be continued for as long as possible [Medicine 2 (BQ)]. In case of serious adverse events, imatinib should be interrupted or reduced to 300 mg/day. Imatinib should be discontinued if serious adverse events continue even after dose reduction, or apparent tumor progression is observed. Although blood concentration measurement can indicate which direction modification of imatinib dose should take, a comprehensive assessment is required when making a decision on dose modification [Medicine 3 (CQ)]. Although retrospective studies suggest a relation between somatic mutation type and PFS, there are no reports supporting the selection of TKIs based on genetic mutations [Medicine 12 (CQ)]. Regardless of gene mutation type, imatinib is recommended as first-line therapy, with sunitinib, regorafenib, and pimitespib subsequently administered in this order if necessary.

##### Medication for imatinib-resistant GIST (Secondary treatment or later)

Sunitinib is recommended for imatinib-resistant GIST [Medicine 6 (BQ)]. Sunitinib demonstrated efficacy in patients with good functional status with an ECOG performance status 0 or 1. The standard regimen is 50 mg/day for 4 weeks on, 2 weeks off. In case of adverse events, the dose is gradually reduced to 37.5 mg/day, 25 mg/day, but the efficacy of doses reduced to below 25 mg/day is unknown. If the standard dosage and administration is intolerable, modification of the administration schedule is an option [Medicine 11 (CQ)]. It should be noted that sunitinib can cause various adverse events such as hand-foot skin reaction, hypertension, malaise, hypothyroidism, proteinuria, and myelosuppression [131]. As with imatinib, regular follow-up and imaging studies are required, and sunitinib should be discontinued if intolerable adverse events or tumor progression is observed.

It should be noted that increasing imatinib dose (> 400 mg/day) in patients resistant to the standard dose is not covered by national health insurance in Japan. Although there is no data for direct comparison of increased dose and sunitinib for patients who are resistant to standard dose imatinib, increased dose has been reported to improve PFS and to be effective in patients with some types of gene mutations, and it is an option in some countries [Medicine 4 (CQ)].

Regorafenib is recommended for sunitinib-resistant patients [Medicine 7 (BQ)]. The standard regimen is 160 mg/day, 3 weeks on, 1 week off. As with sunitinib, regorafenib is indicated in patients with good functional status and regular follow-up is required during administration. Adverse events are also similar to those of sunitinib, but attention should be paid to the risk of serious hepatic disorder. Blood examinations to check liver function should be performed periodically (once/week) for 8 weeks from the start of administration. If serious adverse events occur with the standard dosage and administration schedule, the dose should be reduced to 80 mg/day as the lower limit. Changing the administration schedule may also be an option [Medicine 11 (CQ)]. Rechallenge of TKI for regorafenib-resistant and pimitespib-resistant GIST should be considered if the benefit of administration can be expected to outweigh the harm in individual cases [Medicine 8 (CQ)].

Pimitespib is recommended for regorafenib-resistant patients [Medicine 13 (CQ)]. The standard regimen is 160 mg/day, 5 days on, 2 days off. As with the TKIs for GIST, pimitespib is indicated in patients in good general health and regular follow-up is required during administration. Characteristic adverse events are diarrhea and visual disturbance, and appropriate management such as dose reduction and discontinuation should be initiated considering the results of the RCT for pimitespib.

##### Other treatments

Local treatments, including surgical resection [Surgery 10 (CQ)], radiotherapy [Medicine 9 (CQ)], and transcatheter arterial embolization (TAE) and radiofrequency ablation (RFA) [Medicine 10 (CQ)] for liver lesions, can be a candidate for metastatic GIST [Algorithm 8 (Fig. 8)]. There are no reports which show that survival is prolonged by these local treatments, and the optimal treatment must be chosen for each individual patient. There is little evidence for modification of dosage and administration schedule for sunitinib- and regorafenib-resistant patients, combination of TKIs and local therapy, and treatment of GIST caused by abnormalities other than c-*kit* or *PDGFRA* genes. Since acquiring a comprehensive genomic profile is an option and considering treatment using analytically validated NGS tests, etc., consultations that include experts in sarcoma treatment or admission to high volume centers should be considered on a case by case basis.

#### Adjuvant therapy

The risk of recurrence increases if large tumor diameter, high mitotic counts, or tumor rupture is observed in a completely resected GIST. There is a RCT showing that 3 years of imatinib is better than 1 year in terms of RFS and OS for these GISTs which have a high risk of recurrence [Pathology 5 (BQ)]. At present, 3 years of imatinib therapy is the standard treatment for high-risk GIST [Medicine 5-1 (BQ)]. In addition, the usefulness of imatinib for more than 3 years has not been demonstrated, and future research is needed [Medicine 5-2 (CQ)]. The efficacy of sunitinib and regorafenib as adjuvant therapy has not been demonstrated.

### Questions

#### Medicine 1 (CQ): Is initiating therapy with low-dose imatinib compared to standard-dose useful for metastatic, recurrent, or unresectable GIST for which standard-dose imatinib is indicated?

- **Recommendation:** We recommend not initiating therapy with low-dose imatinib for metastatic, recurrent, or unresectable GIST for which standard-dose imatinib is indicated.
- **Strength of recommendation:** 1 (Strong recommendation)
- **Quality of evidence:** D (Very low quality)
- **Consensus rate:** 92.9%

There is no evidence to suggest there is a benefit in initiating low-dose imatinib for patients who can tolerate standard-dose imatinib (400 mg/day), as there are no reports supporting the efficacy of low-dose imatinib. Since a standard dose or a high dose (600 mg/day) of imatinib as the starting dose has demonstrated its usefulness for metastatic, recurrent, or unresectable GIST in clinical trials, and standard-dose imatinib is to be taken orally once daily after food in adult patients according to the Japanese package insert, we have reached a consensus that it is not appropriate to start at a low dose in patients who can tolerate the standard dose. This recommendation is intended for patients who can start with standard dose. For those who cannot be treated with a standard dose due to their general condition, major organ dysfunction, or adverse events, dose modification from a safety standpoint needs to be considered.

#### Medicine 2 (BQ): Is discontinuation of therapy useful for metastatic, recurrent, or unresectable GIST when tyrosine kinase inhibitors demonstrate efficacy?

- **Recommendation:** We suggest that therapy not be discontinued for metastatic, recurrent, or unresectable GIST when tyrosine kinase inhibitors demonstrate efficacy.
- **Strength of recommendation:** 2 (Weak recommendation)
- **Quality of evidence:** C (Low quality)
- **Consensus rate:** 88.2%

Two RCTs have investigated treatment discontinuation in metastatic, recurrent, or unresectable GIST when imatinib showed efficacy. Both studies showed worsening PFS after discontinuation of imatinib [132, 133]. OS was not significantly different. Both studies had a small sample size (around 50 cases). The differences in adverse events were not reported. Quality of life was reported in one RCT which showed no difference.

It is considered that the harms outweigh the benefits of imatinib discontinuation, but the strength of the evidence was weak because of the small sample size in these RCTs.

We therefore decided “We suggest that therapy not be discontinued for metastatic, recurrent, or unresectable GIST when TKIs demonstrate efficacy.”

Patient preference may vary depending on the balance between toxicity and beneficial effects. No RCT investigating treatment discontinuation was reported for TKIs other than imatinib.

#### Medicine 3 (CQ): Is blood concentration measurement of imatinib useful for metastatic, recurrent, or unresectable GIST?

- **Recommendation:** We suggest that blood concentration measurement of imatinib is carried out for metastatic, recurrent, or unresectable GIST.
- **Strength of recommendation:** 2 (Weak recommendation)
- **Quality of evidence:** D (Very low quality)
- **Consensus rate:** 87.5%

There is no apparent evidence of the usefulness of blood concentration measurement of imatinib administered for metastatic, recurrent, or unresectable GIST. On the other hand, in clinical practice, blood concentration measurement can assist clinical decision-making in limited situations such as re-escalation of imatinib after dose reduction, confirmation of drug compliance, and dose reduction to 200 mg/day or less. In adjuvant therapy, it may be useful in deciding whether to continue or discontinue imatinib treatment or change the dosage, as there are no evaluable lesions for determining drug effects. Although it is difficult to assert that it is useful in all cases of metastatic, recurrent, or unresectable GIST, it might be useful in the situations described above. We consequently suggest that blood concentration measurement should be weakly recommended.

Because the results of blood concentration measurement alone are not sufficient for making clinical decisions, it should be noted that it can be used as one of the factors to be considered in comprehensive clinical decision-making.

#### Medicine 4 (CQ): Is dose escalation useful for metastatic, recurrent, or unresectable GIST which exacerbate at a dose of imatinib 400 mg/day?

- **Recommendation:** We suggest that dose escalation is not carried out for metastatic, recurrent, or unresectable GIST which exacerbate at a dose of imatinib 400 mg/day.
- **Strength of recommendation:** 2 (Weak recommendation)
- **Quality of evidence:** D (Very low quality)
- **Consensus rate:** 94.1%

No RCTs have examined the benefit of dose escalation of imatinib for metastatic, recurrent, or unresectable GIST that progressed while receiving imatinib 400 mg/day, and case–control studies and systematic review articles comparing imatinib to sunitinib were considered [134–137]. Dose escalation of imatinib was inferior to sunitinib in PFS, with no difference in OS. Toxicity profiles were different. Considering the balance of benefits and harms, the harms outweigh the benefits due to inferior PFS compared to sunitinib. Only case–control studies were available, and the strength of evidence was very weak. We suggest that dose escalation is not carried out for metastatic, recurrent, or unresectable GIST which exacerbate at a dose of imatinib 400 mg/day. It should be noted, however, that no comparison has been made between using sunitinib after dose escalation of imatinib and switching to sunitinib from the beginning. In Japan, the use of imatinib at doses higher than 400 mg/day is not covered by insurance for GIST, but the efficacy of imatinib 800 mg/day for GIST harboring c*-kit* exon 9 mutation has been reported [138] and is recommended in global guidelines [46].

#### Medicine 5-1 (BQ): Is adjuvant imatinib therapy for 3 years after complete resection useful for GIST at high risk for recurrence and tumor rupture?

- **Recommendation:** We recommend adjuvant imatinib therapy for 3 years after complete resection is carried out for GIST at high risk for recurrence and tumor rupture.
- **Strength of recommendation:** 1 (Strong recommendation)
- **Quality of evidence:** B (Moderate quality)
- **Consensus rate:** 100%

A high-quality RCT comparing imatinib for 3 years versus 1 year after complete resection in patients with high-risk GIST (modified Fletcher classification, see Pathology 5 (BQ) for risk classification) was reported [115, 139]. PFS and OS were significantly improved with imatinib for 3 years. The improvement in RFS and OS was maintained at long-term follow-up. Grade 3 or higher adverse events increased with imatinib for 3 years.

The strength of evidence is moderate and the benefits are considered to outweigh the harms based on the results of one high-quality RCT. Thus, it was recommended strongly. Patient preference was also consistent with this recommendation.

#### Medicine 5-2 (CQ): Is adjuvant imatinib therapy for more than 3 years after complete resection useful for GIST at high risk for recurrence and tumor rupture?

- **Recommendation:** *Recommendation was not determined even after a second round of voting.
- **Strength of recommendation:** Not Graded
- **Quality of evidence:** D (Very low quality)
- **Consensus rate:** –%

No RCTs have been reported that examined postoperative adjuvant imatinib therapy beyond 3 years after complete resection in patients with high-risk or ruptured GISTs, and only one observational study has been reported [140]. Although this study compared the duration of imatinib for 1 year, 1–3 years, 3–5 years, and > 5 years, a trend toward improvement in OS and PFS was observed with imatinib for more than 3 years. Data on adverse events were not reported.

The level of evidence is very weak, and the balance of benefits and harms is difficult to assess. Although there were two rounds of voting, we were unable to reach a consensus and make a recommendation to either use or not use postoperative adjuvant imatinib therapy beyond 3 years after complete resection. An RCT is currently underway to investigate the significance of imatinib beyond 3 years [46], and the results are awaited.

#### Medicine 6 (BQ): Is sunitinib useful for metastatic, recurrent, or unresectable GIST in patients that are imatinib resistant or intolerant?

- **Recommendation:** We recommend that sunitinib is used for metastatic, recurrent, or unresectable GIST in patients that are imatinib resistant or intolerant.
- **Strength of recommendation:** 1 (Strong recommendation)
- **Quality of evidence:** B (Moderate quality)
- **Consensus rate:** 100%

One high-quality RCT comparing sunitinib with placebo in patients with metastatic, recurrent, or unresectable GIST who had failed to respond to imatinib showed a significant improvement in time to tumor progression with sunitinib, but no significant difference in OS [131]. Because crossover to sunitinib was allowed in the placebo group, RPSFT (rank-preserving structural failure time) analysis was performed and showed a trend toward improvement in OS with sunitinib [141]. Toxicity was increased with sunitinib. The strength of evidence is moderate based on one high-quality RCT. The balance of benefits and harms was considered to be more favorable for sunitinib. Patient preference was also consistent with this recommendation.

Although not included in the recommendation, the efficacy of pazopanib [142] and regorafenib [143] in second-line treatment of GISTs refractory to imatinib has also been reported (not covered by insurance in Japan).

Imatinib-refractory GISTs include those without c-*kit* mutations. For example, the *PDGFRA* D842V mutation is known to be imatinib resistant, and the efficacy of avapritinib has been reported [144] and approved overseas (however, not approved in Japan). The disease is known to be imatinib resistant.

If the patient is intolerant or refractory to standard therapy or does not have c-*kit* or *PDGFRA* mutations, consider performing a comprehensive genomic profiling test such as NGS testing with established analytical validity.

If an NTRK fusion gene is found, entrectinib [145] or larotrectinib [146] is expected to be effective.

#### Medicine 7 (BQ): Is regorafenib useful for metastatic, recurrent, or unresectable GIST in patients who are sunitinib resistant or intolerant?

- **Recommendation:** We recommend that regorafenib is used for metastatic, recurrent, or unresectable GIST in patients who are sunitinib resistant or intolerant.
- **Strength of recommendation:** 1 (Strong recommendation)
- **Quality of evidence:** B (Moderate quality)
- **Consensus rate:** 100%

One RCT as the basis of approval demonstrated the superiority of regorafenib over placebo in PFS for recurrent or unresectable GIST which failed to respond to sunitinib [147]. However, whether the regorafenib group was superior in terms of OS could not be clarified because the patients assigned to the placebo group were crossed over to the regorafenib group after disease progression in this trial. In addition, the long-term result of the preceding phase II trial [148] and the meta-analysis [149] published after the RCT supported the clinical usefulness of regorafenib for recurrent or unresectable GIST after failure to respond to imatinib and sunitinib.

On the other hand, the RIGHT trial also demonstrated the effectiveness of reintroducing imatinib for metastatic, recurrent, or unresectable GIST after failure to respond to imatinib and sunitinib in terms of PFS [150]. However, since no tumor response was observed and few cases experienced a durable anti-tumor effect (median PFS: 1.8 months) in the imatinib group, regorafenib should be administered to patients with metastatic, recurrent, or unresectable GIST after failure to respond to imatinib and sunitinib.

Although there is only one RCT supporting the clinical effectiveness of regorafenib for metastatic, recurrent, or unresectable GIST after failure to respond to sunitinib, the strength of the recommendation was set as “strong” considering the difficulty of conducting the clinical trial due to the rarity of GIST and the situation that no other drug is recommended in Japan.

#### Medicine 8 (CQ): Is rechallenge of imatinib or sunitinib useful for metastatic, recurrent, or unresectable GIST in patients who are regorafenib resistant or intolerant?

- **Recommendation:** We suggest that rechallenge of imatinib or sunitinib is carried out for metastatic, recurrent, or unresectable GIST in patients who are regorafenib resistant or intolerant.
- **Strength of recommendation:** 2 (Weak recommendation)
- **Quality of evidence:** D (Very low quality)
- **Consensus rate:** 94.1%

We did not find any papers showing the efficacy of other TKIs for patients intolerant or refractory to regorafenib. However, the efficacy of imatinib and sunitinib readministration for GIST intolerant/refractory to imatinib and sunitinib has been reported [151, 152]. In addition, the efficacy of imatinib administration has been verified in the RIGHT study [150], which is an RCT of imatinib as standard therapy, including patients previously treated with regorafenib, although the number of cases is small. Since no serious adverse events were reported in the RCT, readministration of imatinib or sunitinib is recommended [153]. However, the strength of the evidence was also very weak, and it was recommended weakly. Pimitespib administration is recommended before readministration because pimitespib is approved as a fourth-line agent.

#### Medicine 9 (CQ): Is radiation therapy useful for metastatic, recurrent, or unresectable GIST?

- **Recommendation:** We suggest that radiation therapy is not used for metastatic, recurrent, or unresectable GIST.
- **Strength of recommendation:** 2 (Weak recommendation)
- **Quality of evidence:** D (Very low quality)
- **Consensus rate:** 94.1%

Although GISTs are not considered radiosensitive, radiotherapy is sometimes used in clinical practice, and the usefulness of radiotherapy for metastatic or recurrent GISTs is an important clinical issue. A review of the articles on the CQ of whether radiotherapy is useful for metastatic GISTs identified two observational studies.

Both were case–control studies, one a multicenter prospective study [154] and the other a single-center retrospective study [155]. The prospective study enrolled 25 patients and examined the efficacy of 30–40 Gy of radiotherapy for hepatic or intra-abdominal lesions of GIST that had progressed during or after TKI therapy. In the retrospective study, the efficacy of radiation therapy was examined in 15 patients with 22 lesions. The study was relatively old, starting in 1997, and included four patients who were not on TKIs, and also varied in terms of lesion location and irradiation dose.

PFS was reported as 4 months in the prospective study and 7.1 months in the retrospective study. TTP at the irradiated site was 16 months in the prospective study. Symptomatic palliation was reported only in the retrospective study, and was reported in 14 of 15 patients. Treatment-related adverse events were reported only in the retrospective study, and the adverse event was grade 3 diarrhea in only 1 of 15 patients. However, only grade 3 or higher adverse events were reported; with no information on grade 2 or lower events. In the prospective study, adverse events were reported, including those unrelated to treatment. Based on these results, we conclude that radiotherapy may be useful for temporary tumor control and symptomatic palliation, but the level of evidence from observational studies alone is very weak due to the wide variation in patient backgrounds. Only a few studies have shown improvement in OS although symptomatic palliation may be expected and there have been no serious adverse events. Thus, regarding the balance of benefits and risks, the benefits do not clearly outweigh the risks, given the cost of treatment and the burden associated with making hospital visits.

Therefore, the strength of the recommendation for this CQ is weak.

#### Medicine 10 (CQ): Is local therapy other than surgical resection useful for metastatic GIST of the liver?

- **Recommendation:** We suggest that local therapy other than surgical resection is carried out for metastatic GIST of the liver.
- **Strength of recommendation:** 2 (Weak recommendation)
- **Quality of evidence:** D (Very low quality)
- **Consensus rate:** 100%

A qualitative systematic review of 8 observational studies was conducted. One of the 8 observational studies was a case–control study [156] and 7 were case series studies [157–163]. The case–control study [156] was a single-center report of TAE with doxorubicin for liver metastases that worsened during treatment with TKIs (imatinib or sunitinib). The study compared doxorubicin transcatheter arterial chemoembolization (TACE) to TKI reintroduction or BSC as historical control for patients with liver metastases that worsened during treatment with doxorubicin (doxorubicin). The instructions for use of doxorubicin differ from those in the Japanese package insert. Of the 7 case series studies, 3 were older ones [157–159] that included sarcomas other than GIST. Four of the 7 studies [160–163] used RFA, 2 used TAE, 1 used TACE, and 1 used TAE or TACE. TAE or TACE was performed in one case. The timing of additional local therapy was reported both after progression and during response to TKIs. The TKIs included imatinib or sunitinib, and were mixed in the same studies. Seven studies were non-Japanese. In terms of PFS, a case–control study reported a longer PFS in the TACE group than in the control group (30 weeks vs. 12.9 weeks). TTP of the locally treated site in the TACE group was reported to be 47.1 weeks. Among the case series studies, four studies included only GIST, of which two studies with RFA did not reach the median duration, one study with TAE reported a median of 4.5 months, and one study with TACE reported a median of 7.0 months. No palliative effect was noted in any of the studies. Most treatment-related adverse events included fever and puncture site pain, with few reports of serious adverse events. Based on the above, the evidence for this CQ is very weak. In some cases, tumor progression can be expected to be suppressed, adverse events may be acceptable, and the benefits may outweigh the disadvantages in situations limited to liver metastases, so the recommendation is “weakly recommended.” It should be noted that the current situation is different from the above evidence due to the clinical introduction of regorafenib, pimitespib, and other drugs. It is also to be noted that it is not clear which patients should be treated or when the patients should be treated, and that pharmacological therapy has to be continued after local therapy.

#### Medicine 11 (CQ): Is alteration of dosage and administration schedule of sunitinib and regorafenib recommended for GIST in patients who are sunitinib- and regorafenib-intolerant at the standard dosage and administration?

- **Recommendation:** We suggest that alteration of the dosage and administration schedule of sunitinib and regorafenib is carried out for GIST in patients who are sunitinib- and regorafenib-intolerant at the standard dosage and administration.
- **Strength of recommendation:** 2 (Weak recommendation)
- **Quality of evidence:** D (Very low quality)
- **Consensus rate:** 94.1%

There were no prospective phase III clinical trials for this CQ, and only one prospective phase III clinical trial for sunitinib [164]. For regorafenib, there was no prospective study, only a retrospective study [165–171].

Both prospective studies and other retrospective studies reported daily administration at reduced doses, but there were no reports that safety and efficacy were significantly impaired. Thus, alteration of dosage and administration schedule is considered to be acceptable when a proper administration schedule is not possible.

However, since there are no factors that can be strongly recommended, we weakly recommend changing the dosing schedule of sunitinib and regorafenib for GIST in patients who are intolerant to standard doses of sunitinib and regorafenib.

#### Medicine 12 (CQ): Is gene analysis useful for choice of tyrosine kinase inhibitors in GIST?

- **Recommendation:** We suggest that gene analysis is not carried out when choosing tyrosine kinase inhibitors in GIST.
- **Strength of recommendation:** 2 (Weak recommendation)
- **Quality of evidence:** D (Very low quality)
- **Consensus rate:** 88.2%

To our knowledge, none of the literature extracted in the screening step addressed genetic analysis for the purpose of drug selection in GIST. Although some studies investigated the relationship between retrospectively checked genetic alteration and the therapeutic effect of single TKI, no evidence or prospective study was detected to answer this clinical question.

However, some studies indicated the potential usefulness of genetic analysis to predict better PFS [172–183]. Therefore, we set the strength of recommendation as above based on the consensus of experts regarding GIST treatment.

#### Medicine 13 (CQ): Is pimitespib useful for metastatic, recurrent, or unresectable GIST in patients who are regorafenib resistant or intolerant?

- **Recommendation:** We recommend that pimitespib is used for metastatic, recurrent, or unresectable GIST in patients who are regorafenib resistant or intolerant.
- **Strength of recommendation:** 1 (Strong recommendation)
- **Quality of evidence:** B (Moderate quality)
- **Consensus rate:** 86.7%

One RCT [184] comparing pimitespib and placebo for metastatic, recurrent, or unresectable GIST in patients who are regorafenib resistant or intolerant and one single-arm prospective study [185] have been reported. In the RCT, the median PFS was significantly prolonged to 2.8 months in the pimitespib group and 1.4 months in the placebo group (hazard ratio 0.51; 95% CI 0.30–0.87, *p* = 0.006). Analysis using the RPSFT (rank-preserving structural failure time) model, which corrects for the crossover bias toward OS, showed a median OS of 13.8 months in the pimitespib group and 7.6 months in the placebo group (hazard ratio 0.42; 95% CI 0.21–0.85, *p* = 0.007), favoring the pimitespib group. Diarrhea was the only Grade 3 or higher adverse event in > 10% of patients, and anemia, renal disorders, malaise, and anorexia were reported in < 10% of patients. Collaborating with the ophthalmology department should be considered when administering pimitespib because of the nonserious visual disturbance characteristic of HSP90 (heat shock protein 90) inhibitors. Since there is one RCT and one single-arm trial, the strength of the evidence is considered to be moderate. Regarding the balance between benefits and harms, the benefits are considered to outweigh the harms. Considering the rarity of GIST, we strongly recommend the use of pimitespib for patients who are regorafenib resistant or intolerant with metastatic, recurrent, or unresectable GIST.

### Supplementary Information

Below is the link to the electronic supplementary material.

**Fig. S1 Supplemental Algorithm 1**, Genotype of GIST. **a** Confirmation of family history and symptoms of NF-1 is recommended before gene analyses. When GIST with SDH gene abnormality is suspected because of gastric origin, juvenile onset, and epithelioid type, SDHB immunohistochemistry may be done first. **b** These genotypes may have multiple GISTs (reference to Supplemental Algorithm 2). P9 means “see Pathology BQ9”. **Fig. S2 Supplemental Algorithm 2**, Differential diagnosis for multiple GISTs. **a** Multiple GISTs associated with NF1 patients usually occur predominantly in the small intestine including duodenum, but are rarely present in the stomach. P10 means “see Pathology BQ10”. (PPTX 59 kb)
