# Supplementary material for: English version of Japanese Clinical Practice Guidelines 2022 for gastrointestinal stromal tumor (GIST) issued by the Japan Society of Clinical Oncology
Source: Int J Clin Oncol. 2024 Apr 13;29(6):647–80. doi: 10.1007/s10147-024-02488-1 (PMC11130037; doi:10.1007/s10147-024-02488-1)
Supplement: Supplementary file 1 — Fig. S1 Supplemental Algorithm 1, Genotype of GIST. a Confirmation of family history and symptoms of NF-1 is recommended before gene analyses. When GIST with SDH gene abnormality is suspected because of gastric origin, juvenile onset, and epithelioid type, SDHB immunohistochemistry may be done first. b These genotypes may have multiple GISTs (reference to Supplemental Algorithm 2). P9 means “see Pathology BQ9”. Fig. S2 Supplemental Algorithm 2, Differential diagnosis for multiple GISTs. a Multiple GISTs associated with NF1 patients usually occur predominantly in the small intestine including duodenum, but are rarely present in the stomach. P10 means “see Pathology BQ10”. (PPTX 59 kb) [file 10147_2024_2488_MOESM1_ESM.pptx]

## Slide 1
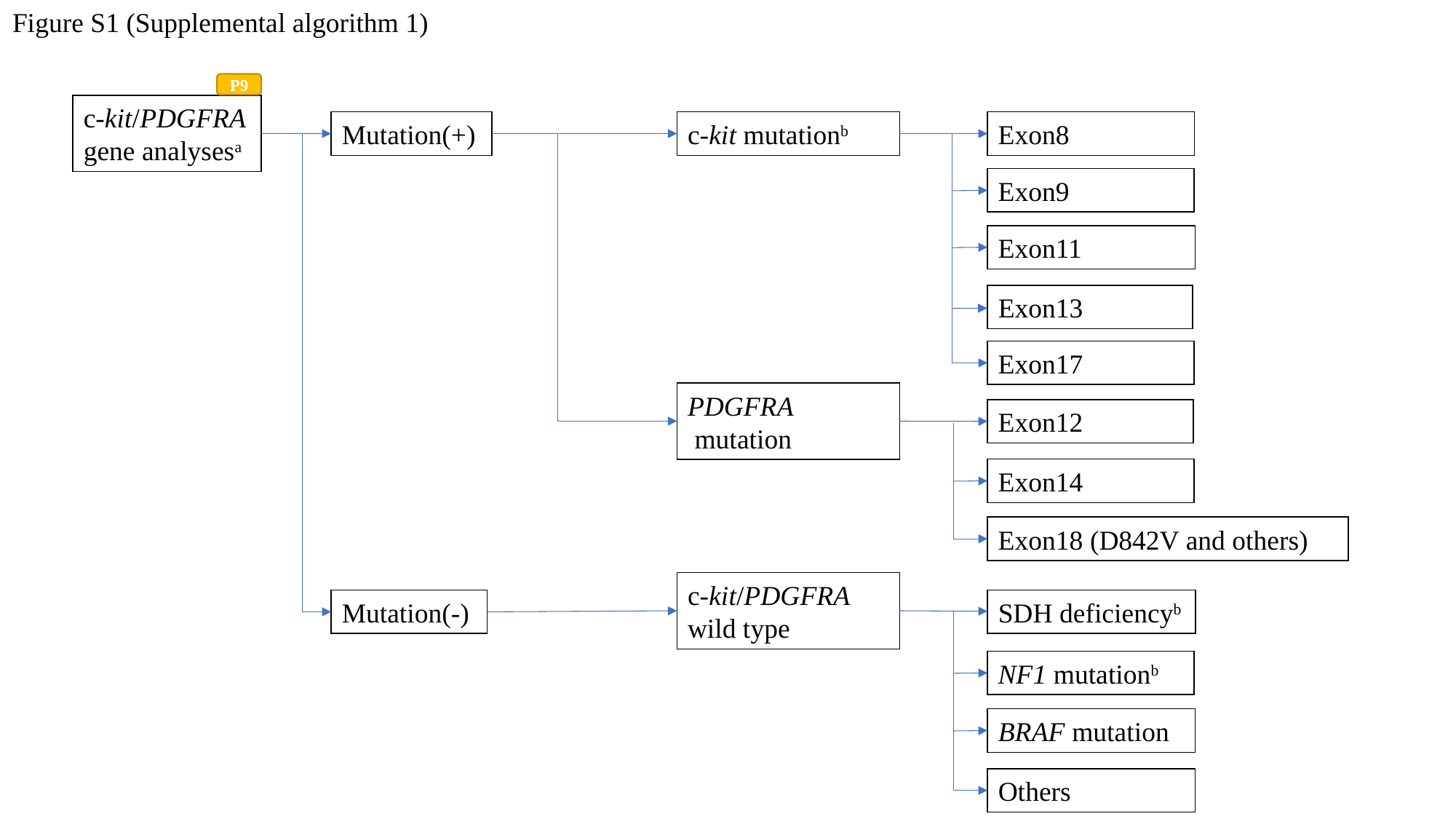

Figure S1 (Supplemental algorithm 1)
P9
c-kit/PDGFRA
gene analysesa
c-kit mutationb
Exon8
Mutation(+)
Exon9
Exon11
Exon13
Exon17
PDGFRA
 mutation
Exon12
Exon14
Exon18 (D842V and others)
c-kit/PDGFRA
wild type
Mutation(-)
SDH deficiencyb
NF1 mutationb
BRAF mutation
Others

## Slide 2
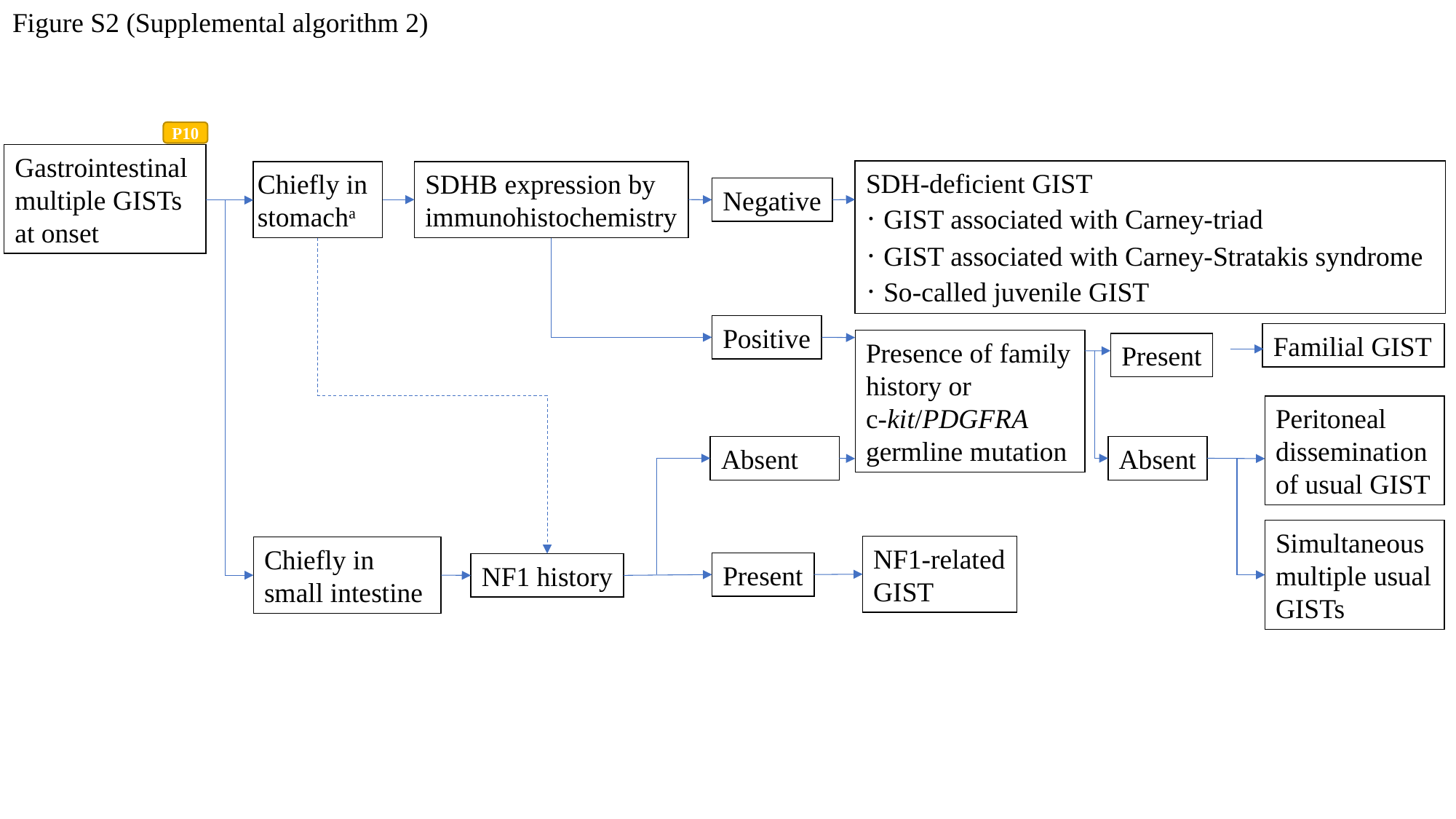

Figure S2 (Supplemental algorithm 2)
P10
Gastrointestinal multiple GISTs at onset
SDH-deficient GIST
· GIST associated with Carney-triad
· GIST associated with Carney-Stratakis syndrome
· So-called juvenile GIST
Chiefly in stomacha
SDHB expression by
immunohistochemistry
Negative
Positive
Familial GIST
Presence of family history or
c-kit/PDGFRA
germline mutation
Present
Peritoneal
dissemination
of usual GIST
Absent
Absent
Simultaneous
multiple usual
GISTs
NF1-related GIST
Chiefly in
small intestine
Present
NF1 history
